# The nucleoplasmic interactions among Lamin A/C-pRB-LAP2α-E2F1 are modulated by dexamethasone

**DOI:** 10.1038/s41598-021-89608-3

**Published:** 2021-05-12

**Authors:** Anastasia Ricci, Sara Orazi, Federica Biancucci, Mauro Magnani, Michele Menotta

**Affiliations:** Department of Biomolecular Sciences, University of Urbino “Carlo Bo”, Via A. Saffi 2, 61029 Urbino, Italy

**Keywords:** Biochemistry, Cell biology, Diseases, Neurodegenerative diseases

## Abstract

Ataxia telangiectasia (AT) is a rare genetic neurodegenerative disease. To date, there is no available cure for the illness, but the use of glucocorticoids has been shown to alleviate the neurological symptoms associated with AT. While studying the effects of dexamethasone (dex) in AT fibroblasts, by chance we observed that the nucleoplasmic Lamin A/C was affected by the drug. In addition to the structural roles of A-type lamins, Lamin A/C has been shown to play a role in the regulation of gene expression and cell cycle progression, and alterations in the LMNA gene is cause of human diseases called laminopathies. Dex was found to improve the nucleoplasmic accumulation of soluble Lamin A/C and was capable of managing the large chromatin Lamin A/C scaffolds contained complex, thus regulating epigenetics in treated cells. In addition, dex modified the interactions of Lamin A/C with its direct partners lamin associated polypeptide (LAP) 2a, Retinoblastoma 1 (pRB) and E2F Transcription Factor 1 (E2F1), regulating local gene expression dependent on E2F1. These effects were differentially observed in both AT and wild type (WT) cells. To our knowledge, this is the first reported evidence of the role of dex in Lamin A/C dynamics in AT cells, and may represent a new area of research regarding the effects of glucocorticoids on AT. Moreover, future investigations could also be extended to healthy subjects or to other pathologies such as laminopathies since glucocorticoids may have other important effects in these contexts as well.

## Introduction

Ataxia Telangiectasia (AT) is a rare autosomal recessive disease caused by the ataxia telangiectasia mutated (ATM) gene^1–3^ encoding for the ATM protein, a large serine/threonine kinase belonging to the PI3 kinase-like kinase (PIKK) family^4^. AT has a prevalence of 1:40.000 to 1:100.000^5^, and it belongs to a premature onset group of childhood ataxias, characterized by neurodegenerative disorders, ataxia, oculocutaneous telangiectasias, immunodeficiency, radio sensitivity and proneness to cancer. The AT disorder has a very complex phenotype, which is probably dependent on the residual kinase activity of ATM^6,7^ and on its amount since this protein has pleiotropic downstream targets, partners and molecular functions^8,9^, in addition to its involvement in double strand breaks (DSB). No cure is currently available for this disease, and typically, AT patients are wheel-chair dependent by the age of ten, and their life expectancy is around twenty-five years. However, several studies have shown that glucocorticoid administration can ameliorate the quality of life and neurological symptoms of AT patients^10–13^.

Studies have been carried out to elucidate the mechanism of action of glucocorticoids in AT cellular models, revealing they can specifically modulate several cellular functions, namely splicing, gene and protein expression, metabolism, red-ox homeostasis, and autophagy^14–21^. While pursuing this line of research, specifically investigating the effects of dexamethasone in AT fibroblasts, we unwittingly observed a variation in the amount of nucleoplasmic Lamin A/C in AT fibroblasts after dexamethasone (a glucocorticoid analogue) administration. A-type lamins are encoded by the LMNA gene and are the main constituents of nuclear lamina, acting as a shell to regulate nuclear shape functions^22,23^. In addition to their purely mechanical function involving their interaction with other nuclear periphery components^24,25^, in the last few years a growing body of evidence has revealed another role of nucleoplasmic lamin. Specifically, Bridger *et al.*^26^ and Hozak et al.^27^ (nucleoplasmic lamin foci and nucleoplasmic lamin filaments respectively) were the first to show that A-type lamins exist in a mobile and low assembly state in the nuclear interior^28,29^. Not only do A-type lamins play a direct role in the chromatin shape modulation, but they are also able to directly influence gene transcription, functioning as an interacting molecular switch. In fact, lamina associated polypeptide (LAP) 2α, a non-membrane bound isoform of the LAP2 family, is able to interact with Lamin A/C in the nuclear interior^30–32^. The two partners, in turn, can interact with the pRB/E2F repressor complex, thus regulating E2F target genes^33–35^. In addition, in the last few years, a double function of LAP2α, as a positive or negative proliferation regulator, has been reported by Vidak et al.^36,37^.

Mutations in the LMNA gene cause several human diseases called laminopathies^38^, and one of the most severe of the laminopathies is the premature aging disease called Hutchinson-Gilford progeria syndrome (HGPS). This pathology shows several cellular defects such as impaired cell signaling and cell cycle regulation, compromised DNA repair and premature senescence^39,40^. The precise molecular mechanisms underlying these cellular defects are under investigation but still unknown. DNA repair impairment and premature ageing are also features of AT^8^.

Against this backdrop, after observing by chance that dex treatment in AT primary cells specifically triggered Lamin A/C nuclear accumulation, we investigated whether glucocorticoid treatment could alter the Lamin A/C mediated cell response in AT and WT. We focused on the overall lamin-genome organization and its relationship with the whole gene expression and subsequently evaluated the local E2F target genes regulated by the Lamin A/C interactors LAP2α-pRB. We were able to show that dex can alter Lamin dynamics and signaling, thus opening a challenging new area of investigation concerning not only cell biochemistry but also the specific role that dex can play in the AT pathology.

## Results

### Dexamethasone increase nucleoplasmic Lamin A/C in primary and immortalized AT cells

**Figure 1:**
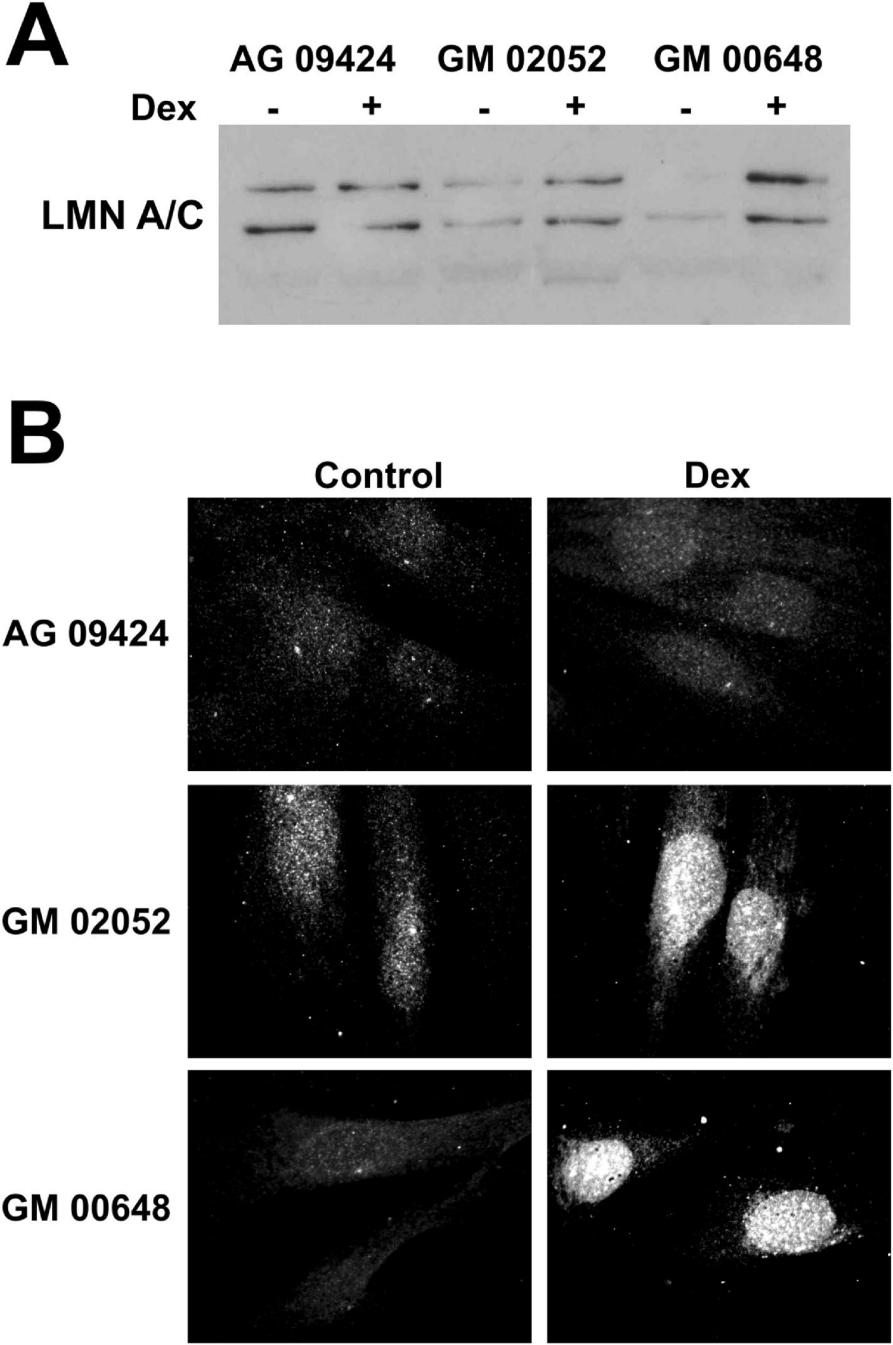
Nuclear accumulation of Lamin A/C. (**A**) Initially used as loading control for western blots of soft RIPA nuclear extracts for another research topic. It then became clear, after repeating the test, that it was not a good housekeeping protein for dex-treated AT primary cells. The amount of Lamin A/C was higher after dex treatment in AT cells, but depended on the cell passage number. Original image S1O in supplementary information. (**B**) Indirect immunofluorescence images produced using Diatheva anti-Lamin A/C on WT and AT primary fibroblasts treated or not with dex for 72 h. The image was obtained from one of the first primary cell passages. In WT, the very low signal stems from the need to avoid CCD saturation when analyzing AT samples.

**Figure 2:**
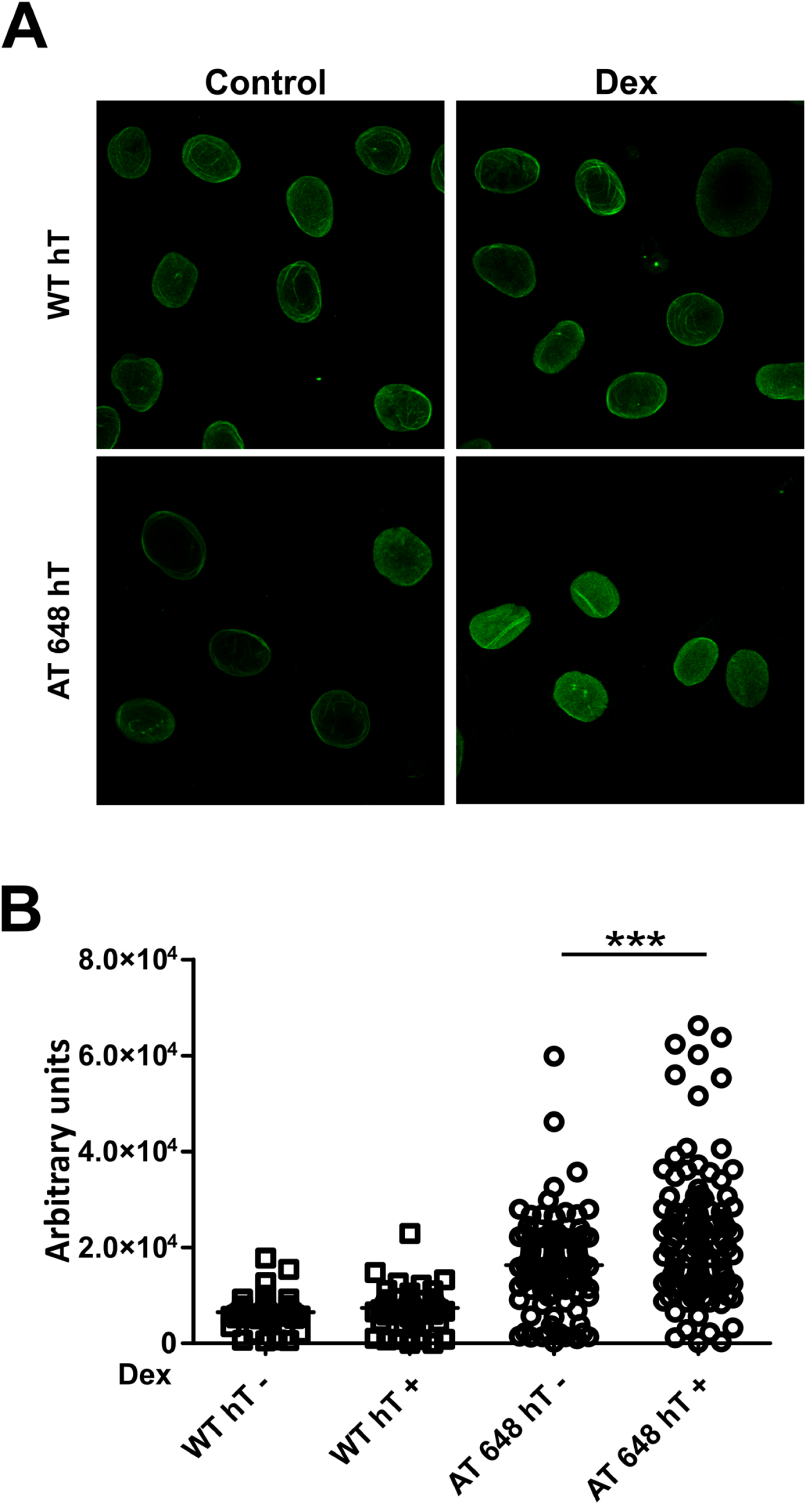
The amount of nucleoplasmic Lamin A/C increased after dex also in immortalized cells. (**A**) Representative images obtained by IF and confocal microscopy of the analyzed WT hT and AT 648 hT samples treated or not with dex for 72 h. (**B**) The nuclear Lamin A/C signals were quantified. On average 300 nuclei were counted and plotted. The increment was statistically significant only in AT 648 hT cells (Welch test, *p* < 0.0001).

**Figure 3:**
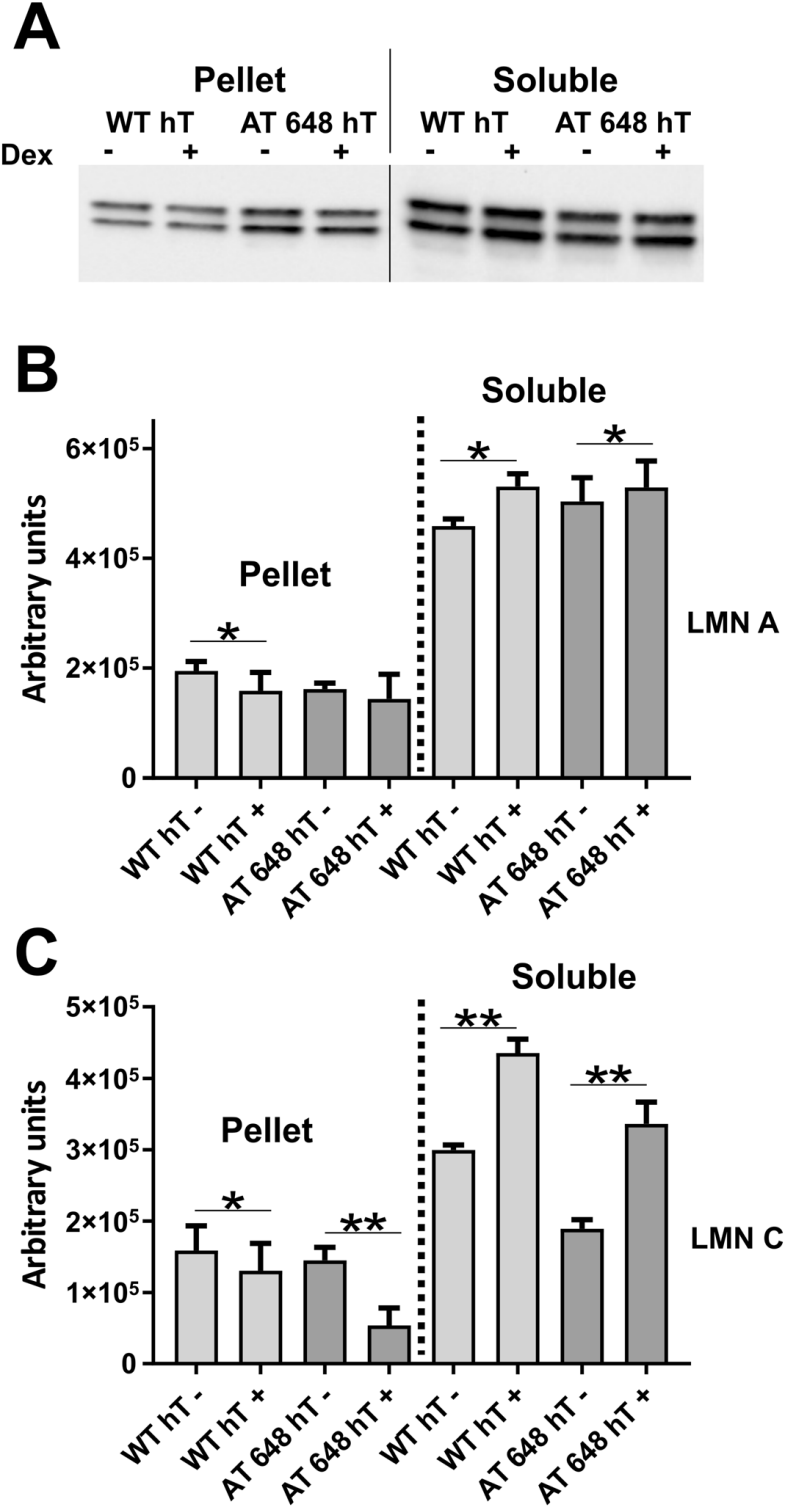
Lamin A/C solubilization assay. (**A**) Illustrative image of the lamins amount in pellets and soluble fractions from WT hT and AT 648 hT cells treated or not with dex. In (**B**) Lamin A and in (**C**) Lamin C quantified amounts. In both samples dex increased the soluble Lamin A (*p* < 0.05, Wilcoxon test n = 4) and Lamin C (*p* < 0.01). Intensities are corrected for loading factor as described in materials and methods. In all samples dex reduced the amounts of insoluble Lamin C (*p* < 0.05 in WT hT and *p* < 0.01 in AT 648 hT) but only Lamin A was altered in WT hT cells (*p* < 0.05). Original images in the series of figures labeled S3O in supplementary information.

Lamin A/C quantification was initially performed as a loading control for western blotting experiments of nuclear protein extracts, obtained from primary WT and AT fibroblasts by gentle extraction procedures, to investigate dex action in primary AT cells. As reported in Fig. 1A, the amount of Lamin A/C was consistently higher in AT cells after dexamethasone treatment. In order to verify the data from the western blots, indirect IF labeling was performed, and the outcome, which confirmed the western blot results, is reported in Fig. 1B. The amount of accumulation was inversely dependent on cell passage numbers. The documented phenotype was therefore tested on derived hTERT immortalized fibroblasts WT hT and AT 648 hT and by using this cellular model, we were able to further confirm the dex dependent nuclear accumulation of Lamin A/C. Although the accumulation was reduced, it was independent of cell passage numbers. Hence, the immortalized cellular model was adopted for all subsequent investigations. The amounts of Lamin A/C in all the tested hT cell lines were inferred by confocal microscopy, as reported in Fig. 2A,B. The WT hT cell presented a smaller quantity of nucleoplasmic Lamin A/C than did the AT 648 hT cells in basal conditions. Dex was able to induce a statistically significant increase in the nucleoplasmic amount of Lamin A/C in AT cells (an average increase of about 50% *p* < 0.001). In order to define the extend of soluble lamin levels of the total nucleoplasmic lamin amount, a solubilization assay was performed as described by Kolb et al.^29^ As illustrated in Fig. 3, the A type lamins were about at 50–60% in a less complex assembly state, possibly containing dimers or short polymers. Dex improved the solubilization process of Lamin A/C in both tested samples (*p* < 0.05) despite its lower amount in WT hT nucleoplasm cells as above stated. Lamin C showed a higher mobility rate than Lamin A (Fig. 3B,C, *p* < 0.01).

**Figure 4:**
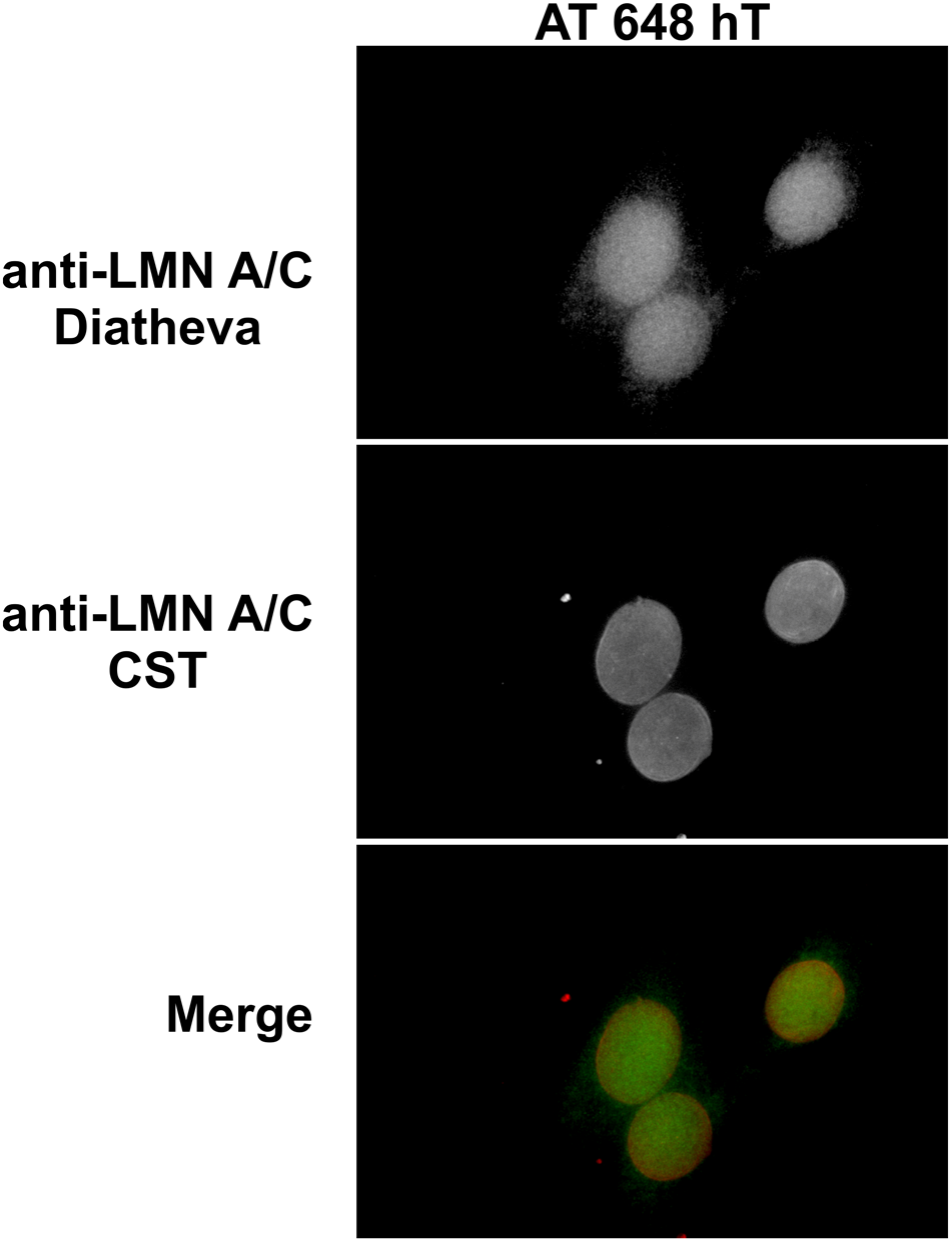
Indirect immunofluorescence images of dex-treated AT 648 hT cells by simultaneously using Diatheva and CST anti-Lamin A/C. Diatheva preferentially labeled nucleoplasmic lamins, while CST also highlighted the nuclear rim.

The amount of Lamin A/C in IF was initially determined using two different antibodies from different suppliers. Although both antibodies led to the same accumulation result, the antibody from the supplier Diatheva seemed to be more sensitive to nucleoplasmic Lamin A/C, while the antibody from the CST supplier was also able highlight the nuclear perimeter (Fig. 4). All investigations were performed by using CST antibody, since it was able to stain all lamin types and only the preliminary results on primary cells were obtained by antibody from Diatheva.

**Figure 5:**
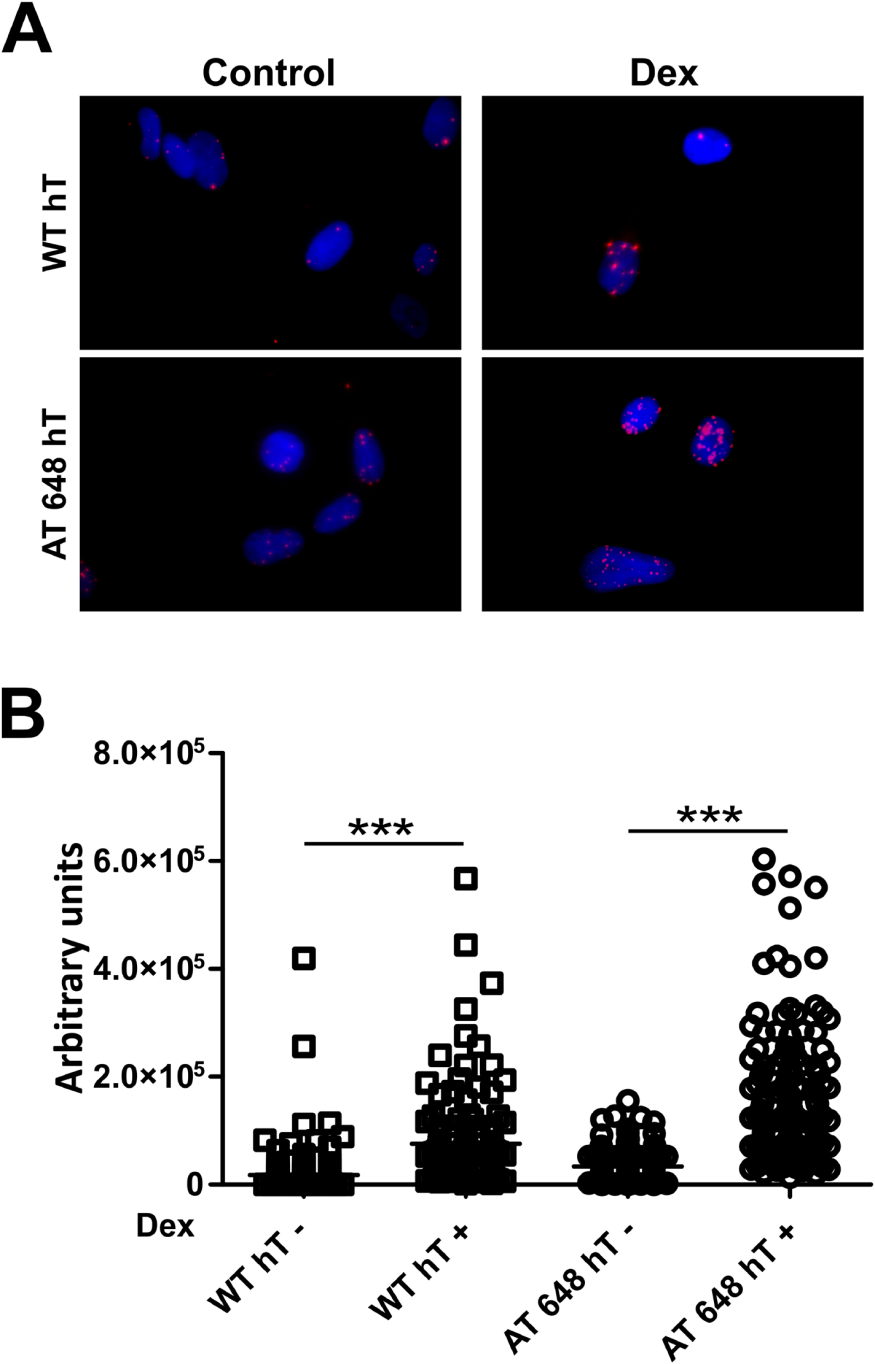
PLA assays revealed that dex promoted Lamin A/C-LAP2α interaction. One of the mechanisms by which Lamin A/C can be solubilized in the nuclear interior is its interaction with LAP2α, and dex was able to increase this interaction both in WT hT and AT 648 hT cells. (**A**) Representative images of PLA assays. The images are DAPI PLA merged. (**B**) Quantification of fluorescence signals; WT hT cells showed a lower basal amount of interaction than AT cells (in accordance with the lower amount of soluble Lamin A/C). On average, there was a four-fold increment in both samples (*p* < 0.0001 Welch test).

**Figure 6:**
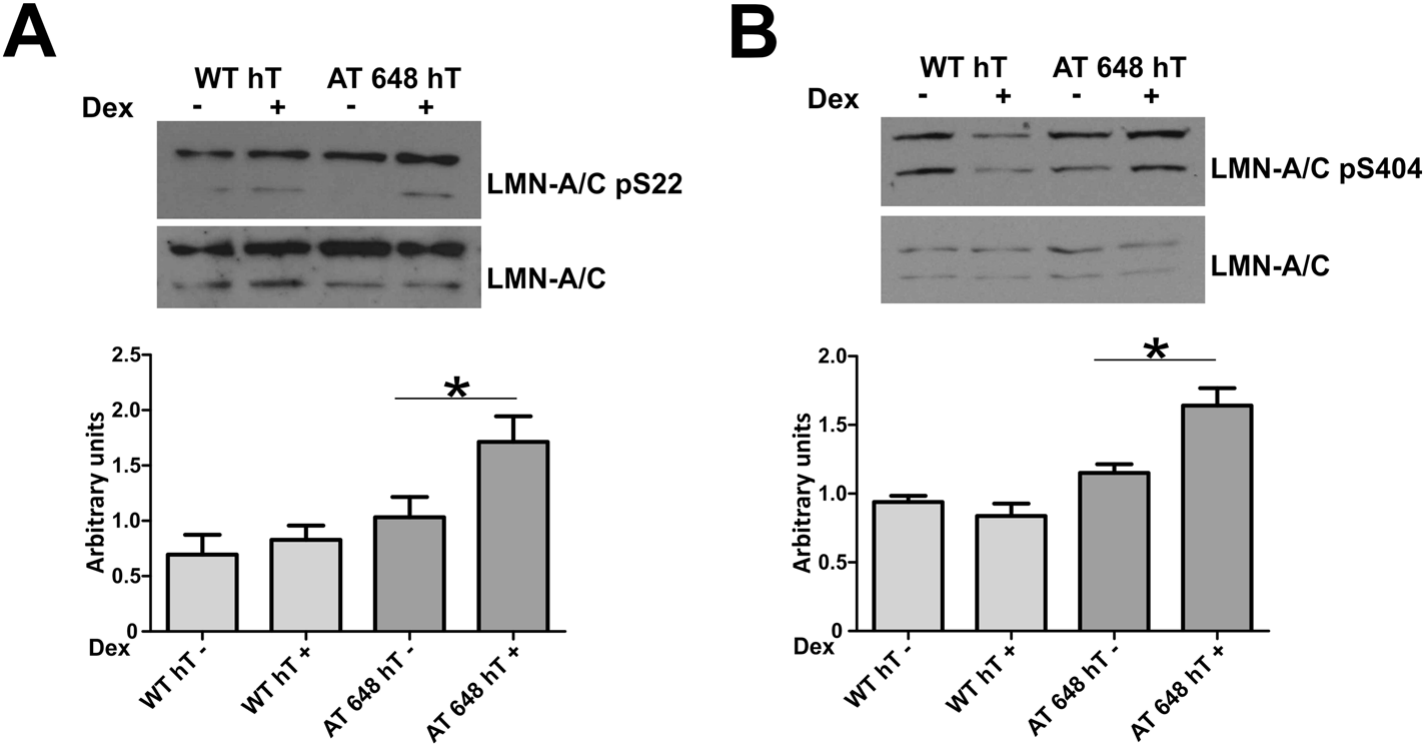
Dex promoted the phosphorylation of S22 and S404 in Lamin A/C. Phosphorylations can participate in A-type lamin A type solubilization. (**A**) Typical western blot by using anti phospho S22 Lamin A/C antibody. Only in AT 648 hT cells was the phosphorylation increment statistically significant (Wilcoxon test *p* < 0.05, n = 7). (**B**) The typical western blot using the anti phospho S404 Lamin A/C antibody. AT 648 hT cells showed a statistically significant phosphorylation increment (Wilcoxon test *p* < 0.05, n = 8). Original images in the series of figures labeled S6O in supplementary information.

Different molecular mechanisms can induce A-type lamin solubilization, and one of the main factors that maintains the Lamin A/C pool in the nucleoplasm is its interaction with the protein LAP2α. We therefore evaluated the effects of dex on this interaction by proximity ligation assay (PLA). As reported in Fig. 5A,B (and in supplementary Fig. S1), dex was able to statistically enhance the Lamin A/C interaction with LAP2α in both WT hT and AT 648 hT cells (*p* < 0.001). The interaction increase was consistently more elevated in AT 648 hT cells than in WT cells. Lamin A/C nucleoplasm accumulation and solubilization may also be due to two independent phosphorylations. One of the phosphorylations usually acts during cell division in S22^41^, while the other acts in S404^42^. We tested both possibilities by western blot (Fig. 6A,B) and serine residues were similarly and statistically more phosphorylated only in AT 648 hT cells after dex treatment (*p* < 0.05). Notably, using whole protein extraction in the strong denaturating conditions used for western blotting, the amount of total A-type lamins was almost the same in both samples, thus confirming that the drug was able to change the mobility status of Lamin A/C but not its amount. Taken together, all the mechanisms described above may contribute to the Lamin A/C solubilization and nucleoplasmic accumulation.

### Lamin A/C chromatin binding is modulated by dex

Recent reports on the role of lamins in other cell models^34,43,44^, suggest that A-type lamins may also associate with chromatin in the nuclear interior, thus regulating whole gene expression. Hence, after having tested the probable mechanism of action of nuclear lamin accumulation, we turned our attention to this possible association. We performed ChIP for Lamin A/C. Tester and respective control samples were analyzed by deep sequencing. The enrichment domain detector (EDD) peak-calling algorithm^45^ allowed us to identify 2244 and 1656 EDD enriched domains in dex-treated WT hT and AT 648 hT samples respectively, with an average length (medians) of 80 and 90 kbp ranging to over 500 kbp as reported in supplementary Fig. S2. In order to identify the genes potentially influenced by chromatin/A-type lamin binding, we recovered them from the regions enclosed and surrounding (+—10kbp) the retrieved genomic intervals. 3590 and 5448 gene symbols were retrieved in dex-treated AT 648 hT and WT hT samples respectively (supplementary file S1). The dex treated samples had 387 shared EDD calls containing 745 gene symbols. These data suggest that dex could alter Lamin A/C -chromatin binding in both WT and AT samples though in different ways.

### Microarray data analysis and integration with Lamin A/C-chromatin binding

**Figure 7:**
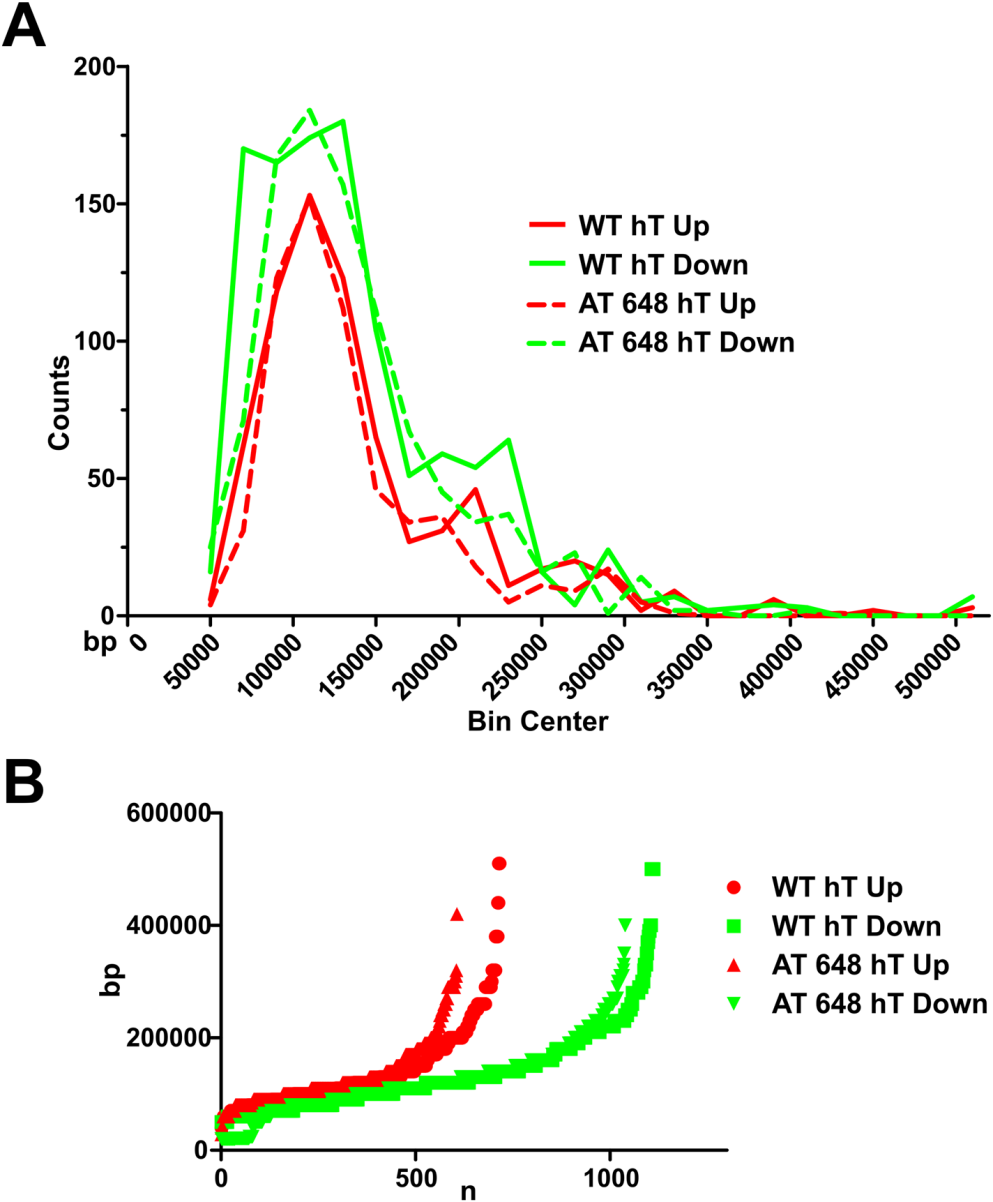
Relationship between EDD peak size and enclosed dex up- or downregulated genes. Dex modified the binding of A-type lamin to chromatin and altered the gene expression of treated fibroblasts. (**A**) Up (red) or down (green) gene expression variation was independent of the binding size of lamin to chromatin in both WT and AT cells since the graphs are mostly overlapping. (**B**) A larger amount of EDD regions preferentially matched with downregulated genes. This evidence is consistent with the current consensus that large Lamin A/C complexes bind heterochromatin.

The matching between overall gene expression variations and Lamin A/C chromatin binding distribution after dex action was then carried out. Gene expression analysis was performed by microarray technology. Using the adopted DEG selection criteria, it was possible to identify 5901 upregulated and 4638 downregulated gene symbols in dex-treated WT hT samples, while in dex-treated AT 648 hT samples, a total of 5595 upregulated and 7150 downregulated gene symbols were identified (supplementary file S2). The biological and molecular functions of the analyzed gene sets were retrieved (Reactome FI networks, plots in supplementary Fig. S3) and are summarized in Table 1, while the full biological processes (BPs) and pathway enrichments of the networks are reported in supplementary file S3. As previously stated in other published papers by our group, we observed differences in how dex acted in regulating gene expression in healthy and AT cells, including fibroblast cells. Consequently, the resulting biomolecular pathways modulated by the drug were also different. We subsequently verified the possible pairing of the whole gene expression with the size of the detected EDD genomic regions, testing whether the up- or downregulated genes were dependent on the length of the Lamin A/C genome binding. The genes highlighted by microarray (subdivided into up- and downregulated genes) were therefore matched to those obtained by EDD region gene fetching and the genomic intervals were retrieved. The output of the analysis is illustrated in Fig. 7. The graphs show a similar pattern (Fig. 7A) and the gene expression appears to be independent of the extent of the Lamin A/C binding in the corresponding gene location. There was only a slight difference between WT hT and AT 648 hT downregulated genes, in the 50–80 kbp range, and a minor difference in both down- and upregulated genes in the 200 kbp region between the WT hT and AT 648 hT datasets. Notably, different quantities of EDD calls were matched to up- and downregulated genes. In fact, in both WT and AT samples, a greater number of calls were related to downregulated genes (Fig. 7B). Specifically, in WT cells, 716 and 1112 calls matched with up- and downregulated genes respectively, while in AT cells, 606 and 1039 calls matched with up- and downregulated genes respectively.

**Table 1:** Reactome FI network clusters extrapolated by gene lists obtained by microarray analysis.

WT hT	Module #	AT 648 hT
Regulation of transcription	0	Regulation of transcription
Phosphatidylinositol- phosphorylation signal transduction	1	Phosphatidylinositol- phosphorylation signal transduction
Cell division- mitotic cell cycle	2	Cell adhesion
Cytokine-mediated signaling pathway	3	SWH pathway
Cell adhesion	4	G protein-coupled receptor signaling
Cellular protein metabolic process	5	DNA repair
Detection of chemical stimulus-G protein-coupled receptor signaling pathway	6	Cell cycle
Protein ubiquitination	7	Detection of chemical stimulus-G protein-coupled receptor signaling pathway
Xenobiotic metabolic process	8	mRNA splicing, via spliceosome
mRNA splicing, via spliceosome	9	GTPase mediated signal transduction
Cornification- cellular protein metabolic process	10	Vesicle docking
DNA replication- DNA repair	11	Macroautophagy
Calcium homeostasis-signaling	12	Cytoskeleton organization
Cytoskeleton organization and signaling	13	Vesicle-mediated transport
Complement activation	14	Glutathione metabolic process
Vesicle docking	15	
Regulation of cell shape and axonogenesis	16	
Macroautophagy	17	
Cell adhesion- SWH pathway	18	
Wnt signaling pathway	19

### Local Lamin A/C interactors and gene regulation

**Figure 8:**
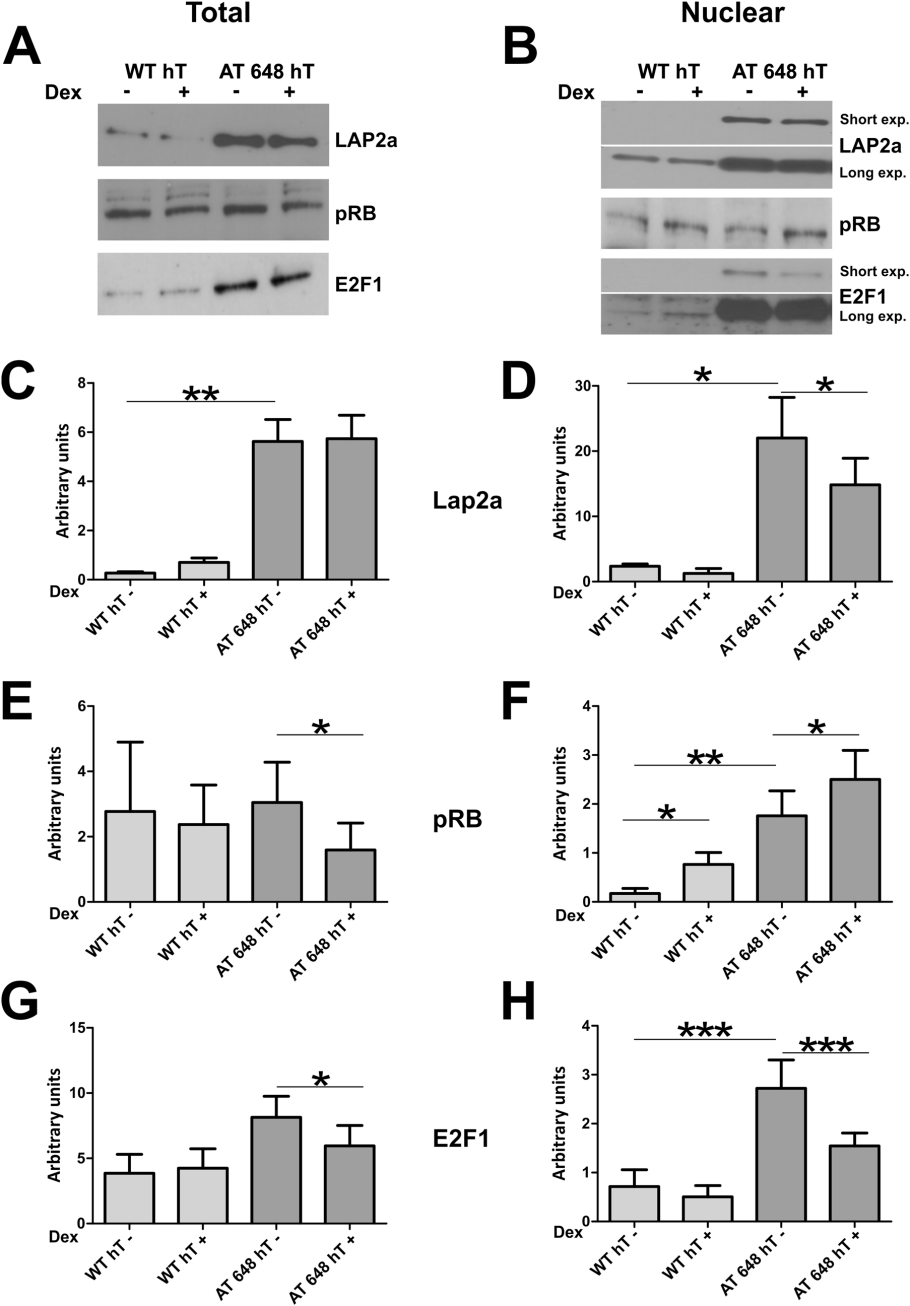
The cellular quantities of LLRE complex components are dex modulated. (**A**) Typical western blots of total E2F1, pRB and LAP2α protein amounts. (**C**) AT cells showed a larger amount of LAP2α than WT (Mann–Whitney test *p* < 0.01, n = 8). Dex altered the pRB (in **E**) and E2F1 (in **G**) amounts only in AT 648 hT sample (Wilcoxon test *p* < 0.05, n = 8). (**B**) Representative western blots of nuclear E2F1, pRB and LAP2α. (**D**) Also in the nuclear compartment LAP2α is more abundant in the AT sample than in the WT sample (Mann–Whitney test *p* < 0.05, n = 8), and dex reduced the amount of LAP2α only in AT 648 hT cells (Wilcoxon test *p* < 0.05, n = 8). (**F**) Nuclear pRB was higher in AT than in the WT cell line (Mann–Whitney test *p* < 0.01, n = 8). Dex increased the nuclear amount of pRB in both samples (Wilcoxon test, *p* < 0.05 n = 8). (**H**) The nuclear amount of E2F1 was more elevated in AT than WT cells (Mann–Whitney test *p* < 0.001, n = 8), and dex considerably reduced its quantity only in AT 648 hT cells (Wilcoxon test, *p* < 0.001 n = 8). Original images in the series of figures labeled S8O in supplementary information.

In order to gain insight into the possible role of Lamin A/C accumulated after dex treatment in AT cells we focused our analysis on this possible role at a lower genetic level. One of the most studied functions of Lamin A/C in gene control is its role in regulating the transcription factor E2F1 by establishing a dynamic interaction with the partners of the complex, LAP2α and pRB. The total and nuclear amounts of these proteins were evaluated before and after dex treatment by western blotting. Figure 8A,B show representative images of the total and nuclear amounts of the tested targets, respectively. The corresponding quantifications are illustrated in Fig. 8C–H. AT cells expressed a larger amount of LAP2a (*p* < 0.01) and its nuclear quantity was also more elevated than it was in WT (*p* < 0.5, in C and D). Moreover, dex was capable of reducing its nuclear localization statistically only in AT (*p* < 0.05). The expression of the corresponding gene (TMPO) was downregulated in both samples. The total quantity of the pRB protein was downregulated by dex only in the AT 648 hT sample (in E, *p* < 0.05), while the nuclear amount was higher in AT 648 hT than it was in WT hT under basal conditions (*p* < 0.01). On the other hand, dex increased the nuclear amount of pRB in both WT hT and AT 648 hT (in F, *p* < 0.05). The matching gene expression was not affected by the drug in either of the samples. The total amount of E2F1 was decreased by dex only in AT 648 hT (in G, *p* < 0.05), while the E2F1 in the nuclear compartment was considerably more elevated in AT cells than in WT cells (*p* < 0.001), and dex reversed this condition (in H, *p* < 0.001). E2F1 gene expression was not regulated by the drug.

**Figure 9:**
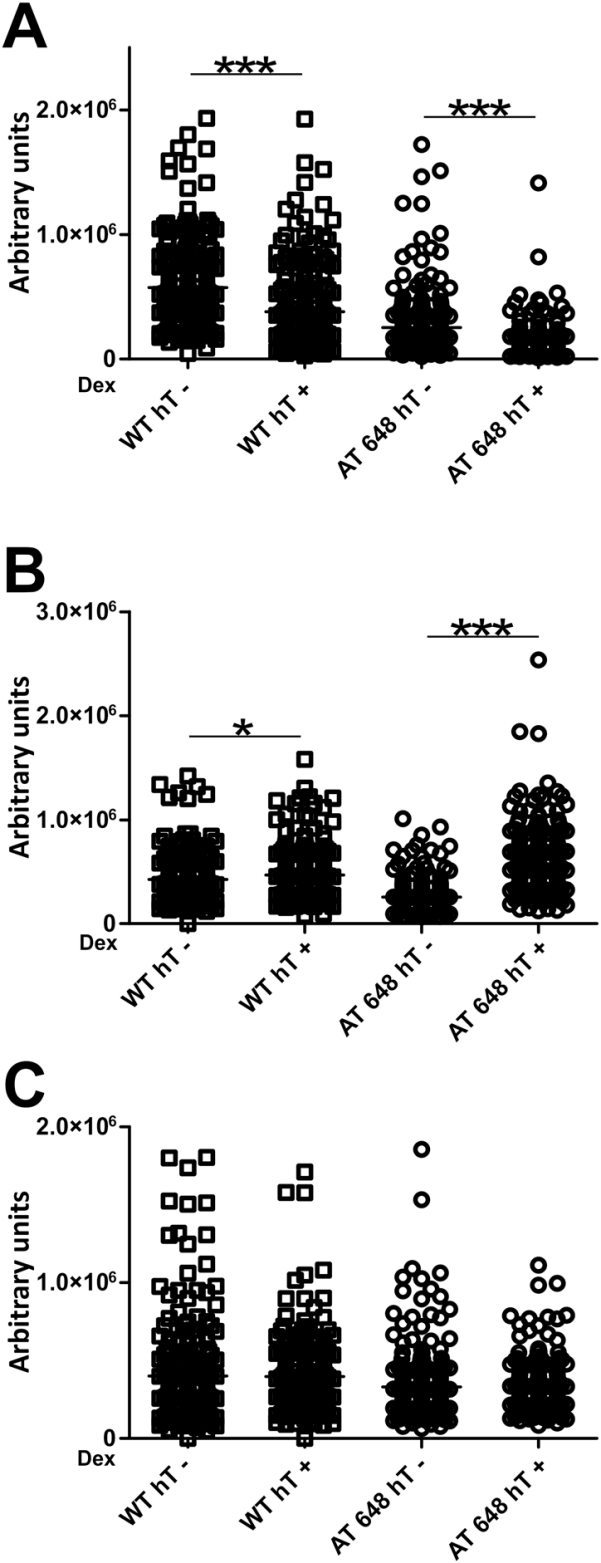
PLA assay outcomes showed that dex altered the interactions between the LLRE elements. (**A**) Dex decreased the extent of nuclear interaction between Lamin A/C and pRB both in WT hT and AT 648 hT cells (Welch test *p* < 0.001). (**B**) Dex increased the extent of nuclear interaction between Lamin A/C and E2F1 in the WT sample (Welch test *p* < 0.05) and in the AT 648 hT sample (Welch test *p* < 0.001). (**C**) No changes were detected in the interaction between pRB and E2F1.

The possible interactions between the partners and the effects of dex in altering these dynamic interactions were then evaluated by PLA. Representative images of the PLA assays are reported in supplementary Fig. S4-6. The signal quantifications are illustrated in Fig. 9. All the interactions were assessed as previously reported in the literature and dex was found to be capable of influencing those interactions as described for Lamin A/C-LAP2α. The Lamin-pRB (A) interaction was decreased by dex in both WT and AT samples (*p* < 0.001) with a large reduction in AT 648 hT. In addition, the E2F1-Lamin (B) interaction increased in both samples, but to a larger extent in AT 648 hT (*p* < 0.05 in WT and *p* < 0001 in AT), while no modulation was statistically recorded for the pRB-E2F1 tests (C). Taken together, these results show that the well-known interacting complex LMN-LAP2α-pRB-E2F1(abbreviated in LLRE) can be dex modulated, and consequently the downstream gene expression as well.

Focusing on the involvement of these interactions in specific gene regulation we turned our attention to two chosen E2F1 regulated genes. We used TFactS, a software that predicts which transcription factors are regulated, inhibited or activated in a biological system, based on lists of upregulated and downregulated genes generated in microarray experiments. We selected BRAC1, a gene that is only downregulated in AT648 hT, and THBS1, which, on the contrary, is only upregulated in AT. Neither of the genes was listed in supplementary file S1, thus they were likely lamins epigenetic role independent.

**Figure 10:**
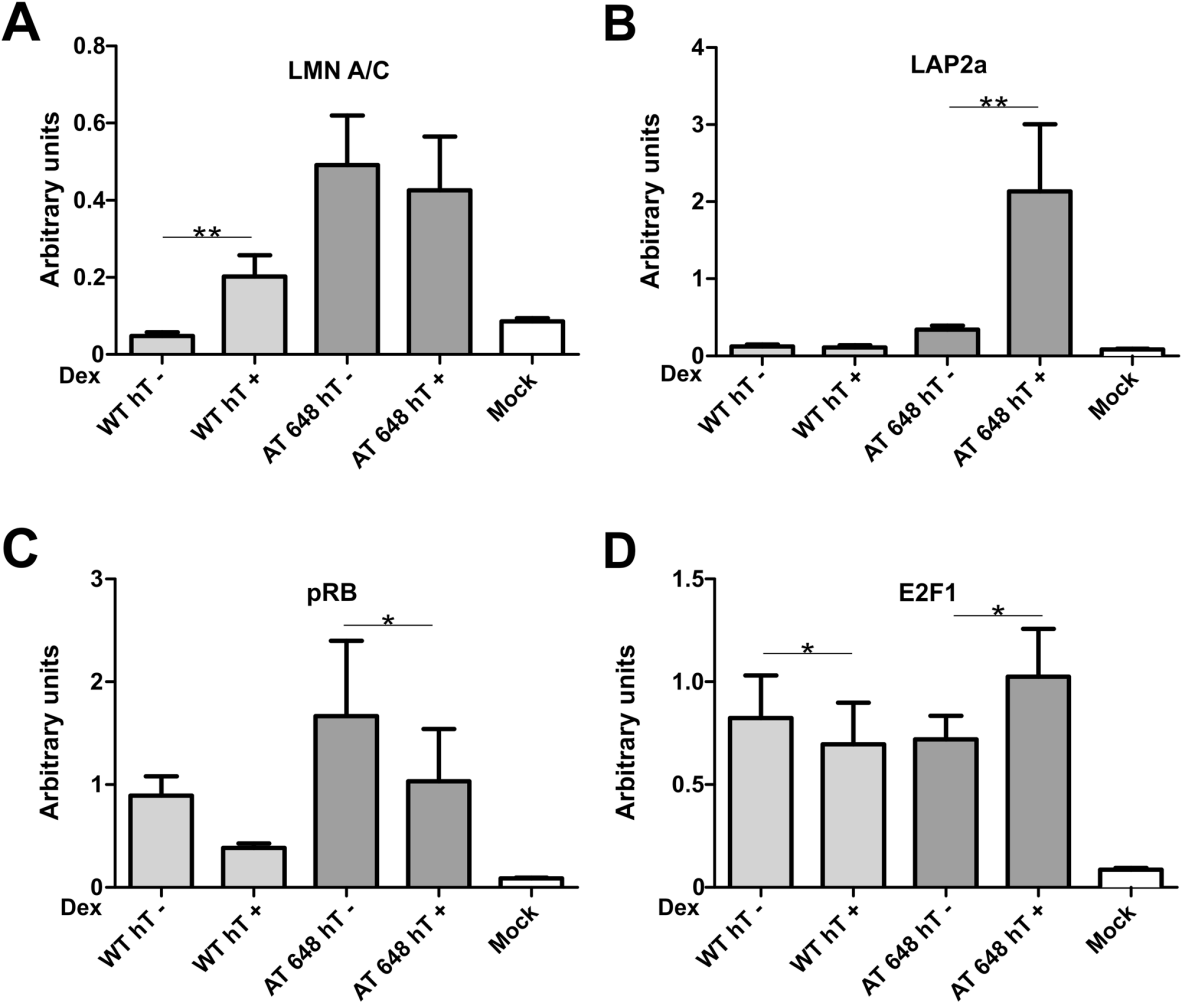
LLRE complex composition on E2F1 binding site of THBS1 promoter. THBS1 is specifically upregulated by dex only in AT 648 hT cells and likely transcribed by E2F1. In the E2F1 consensus binding site the amounts of (**A**) Lamin A/C, (**B**) LAP2α, (**C**) pRB and (**D**) E2F1 are reported for all samples. The E2F1 improved transcription of THBS1 only in AT 648 hT. This improvement seemed to be dependent on the increase in E2F1 itself, an increase in LAP2α and a concomitant decrease in pRB.

**Figure 11:**
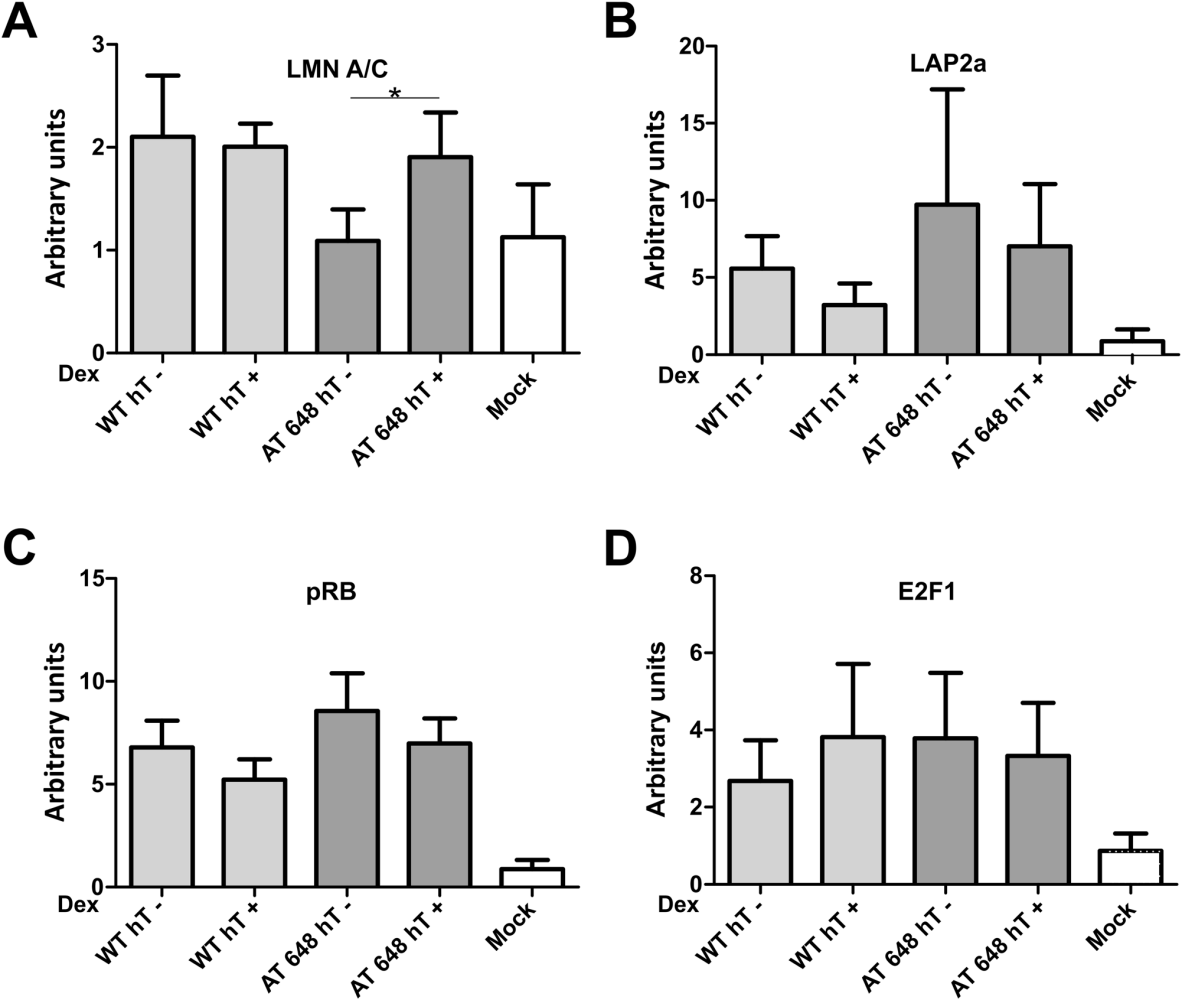
LLRE complex composition at the E2F1 binding site of the BRCA1 promoter. BRCA1 is specifically downregulated by dex only in AT 648 hT cells, and likely transcribed by E2F1. At the E2F1 consensus binding site the amounts of (**A**) Lamin A/C, (**B**) LAP2α, (**C**) pRB and (**D**) E2F1 are reported for all samples. In this context, the only reasonable explanation is the inhibitory repressor effect of Lamin A/C on the LLRE complex, while all other factors remain unchanged.

The direct relationship between gene expression and E2F1 partner regulation was assessed by ChIP using all the antibodies against LLRE components, followed by qPCR analysis of the fragment surrounding the E2F1 binding site in the promoters of the above-mentioned genes. The amounts of the proteins localized in the promoters are shown in Fig. 10 (THBS1) and Fig. 11 (BRAC1). In the BRCA1 promoter, the only difference observed was the significant increase in the amount of Lamin A/C in treated AT 648 hT cells (*p* < 0.05). The graphically observable reductions in Lap2α and pRB were not statistically significant. Regarding THBS1, the quantity of lamins increased in the treated WT sample promoter (*p* < 0.01), the amount of Lap2α increased only in treated AT 648 hT (*p* < 0.01) with a concomitant reduction in pRB (*p* < 0.05), and E2F1 showed a statistically significant decrease in WT, while it increased in AT (*p* < 0.05) following treatment.

## Discussion

The present investigation is the product of an accidental discovery made when Lamin A/C was used as a housekeeping protein to normalize western blot signals for the relative quantification of the nuclear proteins. Surprisingly, Lamin A/C was a bad target to normalize signals since it was upregulated after dex treatment in primary cells. Since during the investigation we adopted a weak method for protein extraction, we deduced that the extracted lamins were in soluble form, as previously described^29,46^.

We further investigated the issue by microscopy and different kinds of antibodies, concluding that the increase in lamins in the nucleoplasm accounted for the findings obtained with western blots. Since the phenomenon was cell passage number dependent, we refocused our research on immortalized cells, previously used in other investigations^47^. An increase in nucleoplasmic lamins, albeit a smaller one, was also observed in this new cellular modal regardless of cell passage numbers. These results suggest that cell senescence may contribute to the observed phenotype. The total amount of lamins persisted unaltered (indeed, a slight but statistically insignificant decrease was observed), for at least 72 h after treatment. We should also bear in mind that the different procedures that were used for total and nuclear protein extractions might contribute to these results. In fact, it is likely that the strong denaturing whole cell lysis also dissolved the structural lamins in the cell ring and inner scaffolds, while for the nuclear fraction, some structural lamins might have been lost in the debris because of the softer extraction buffer. The amount of little assembled lamins was quantified by a solubilization assay that confirmed the increment of unstructured A type lamins. As previously reported in literature^29^ the C type lamin showed a higher mobility than A type, and it was much more soluble after dex treatment in AT cells. Dex was indeed capable to increment the nucleoplasmic lamin in soluble form, this indication was observed both in WT hT and AT 648 hT cells, with less extent in WT especially for the Lamin C. The non-statistical decrease of Lamin A in AT pellets suggested that a fraction of Lamin was not easily extracted by detergent compared to WT. Taken together these indications indicate that probably in AT the incremented nucleoplasmic Lamin A/C existed in both soluble and less solvable forms, the lasts may be caused by differential assembly not present in WT hT samples.

The complexity of lamin arrangement was also supported by the results obtained by IF microscopy using two different antibodies, which allowed us to observe dispersed Lamin A/C in the nucleus. The CST antibody preferentially recognizes the lamin C-terminal, generally referred to as lamin, involved in forming higher-order chromatin structures by various interactions, especially with polycomb (PcG) complexes^43,48–52^. These complexes are generally associated with heterochromatin regions such as the nuclear ring, which was well stained in our experiments, or in the inner foci of nuclei. The Diatheva antibody recognized the full length lamin and therefore also the N-terminal, which is generally associated with the more soluble portion of lamins in the euchromatic regions, thus probably liable diffused Lamin A/C signal increment detected in the proposed cellular model.

The amounts of A type lamins in nuclear rims were also quantified (not shown) and within the measurement possibility limit, we did not observed variations. Anyway, a deeper investigation is necessary to assess this matter in more accurate manner, with certain regard to the interaction with other lamin types.

To our knowledge, this is the first report to show that glucocorticoids can act on Lamin A/C dynamics. Nayebosadri et al. previously reported a relationship between lamins and glucocorticoids^53,54^, with particular interest in GC receptors, but in completely different experimental conditions and without citing any evidence regarding the effects of dex on nucleoplasmic lamins. We therefore believe that the present investigation may pave the way for a new branch of research concerning the effects of glucocorticoids on Lamin A/C and all downstream related effects. We have shown that the solubilization of lamins and their localization in the nucleoplasm are probably due to three concomitant events, namely the phosphorylation of two residues and the interaction with the partner LAP2α. Notably, it has been reported that phosphorylation in Ser404 is a downstream process mediated by AKT activation^42^, and we have previously shown that AKT is strongly activated by dex^47^ and probably triggered the Ser404 modification.

We then sought to determine the consequences of the observed changes in the Lamin A/C dynamics in the nuclear interior. It has been shown that LAP2α-Lamin A/C complexes act on chromatin modelling and thus play a role in epigenetics and indirect gene expression regulation. We used a specific bio-informatic approach, specifically designed for broad enrichment domains of ChIP-seq data, using the lamin antibody. We found that dex was able to alter the binding capacity of Lamin A/C with chromatin. The way that the binding capacity was altered varied between WT and AT samples. In any case, the number of counted domains was surprising. In fact, we had expected a larger number of EDD calls in the AT samples, due to the larger amount of nucleoplasmic Lamin A/C, yet fewer interacting regions were calculated. It is therefore likely that dex promoted the release of Lamin A/C from large chromatin complexes (LADs lamina-associated domain), measured as peak by EDD, promoting smaller Lamin A/C interacting regions or non-interacting Lamin A/C monomers. The peak calls were higher in WT cells, where a small amount of nucleoplasmic lamins were measured, and lower in the AT samples, where a large amount of soluble A-type lamins were detected. Moreover, the Lamin C mobility was particularly different in AT, and its distinct role in ChIP procedure cannot be excluded.

The complexity of LADs in the functional organization of the genome was recently thoroughly reviewed by Briand and Collas^55^. In the scenario described by these authors, the dynamic interconnection of several factors such as Polycomb proteins, Histones, HDACs and other scaffold proteins can be the source of Lamin A/C release with subsequent gene regulation in both hetero and euchromatin regions. It has been reported that phosphorylated Ser22 A-type lamin (soluble lamin, as reported by the authors) binds to active enhancers in premature aging syndrome^56^. Moreover, we cannot exclude its function in the gene regulation of non-pathogenic conditions, an important topic that warrants further investigation. The effects of dex-Lamin A/C chromatin remodeling on whole gene expression revealed that dex affected WT and AT cells differently. The result regarding AT cells was in agreement with our published paper concerning transcriptome analysis^20^ (and Ricci et al., submitted paper). Furthermore, in the present investigation, in the cellular AT model, dex influenced several AT impaired pathways, including the DNA repair pathway.

The relationship between the overall gene expression and Lamin A/C chromatin regulation in dex-treated samples was inferred by matching microarray data with ChIP-seq data. The association between the EDD call size in the genome and the enclosed up- or downregulated genes seemed constant, suggesting that the down or overexpression of enclosed genes was independent of the binding size. However, the gene symbols from the transcriptome analysis, like those contained in EDD calls, were preferentially downregulated genes. This supports the idea that the EDD calls preferentially belonged to the heterochromatin domains, while a portion, representing about the 30–40% of the calls, was associated with euchromatin. The global gene expression variation showed an up-down regulated gene ratio of about 1.1 in WT and 0.6 in AT. Clearly, other factors at higher or lower levels may contribute to gene expression regulation, and lamins only partially correlate with the global annotation, but the role of A-type lamin in euchromatin in the nucleoplasm can also exist at lower levels. Among the contributing factors, the released soluble Lamin A/C by dex acts as a direct gene regulator through the previously described LAP2α-Lamin A/C complexes (Foisner group), in regulating pRB and E2F transcription factors. Indeed, several mechanisms have been proposed to explain how nucleoplasmic Lamin A/C and LAP2α-affect pRb-E2F functions^33,57–62^. When we focused on these factors, we observed that dex was able to modulate their nuclear amounts and their reciprocal interactions, suggesting that the downstream effectors were also altered.

Firstly, AT cells showed an unbalanced nuclear and total amount of the proteins Lamin A/C, LAP2α, pRB and E2F in basal conditions. Such a condition might be associated with ATM deficiency. The crosstalk between ATM and pRB-E2F1 has been described in the literature as controlling cell cycle progression and apoptosis^63–65^ pathways possibly through ATM kinase activity, the lack of which might contribute to the observed quantities and redistribution of these factors in AT cells. No evidence has previously been reported for LAP2α in the AT condition showing a divergent pattern from WT cells. This may be due to the behavior of the above-mentioned proteins, but also to the reported distinctive Lamin A/C dynamic in AT cells. Notably, the amount of LAP2α in basal conditions in AT cells correlated with a greater number of interactions with lamins. This did not occur in WT cells. Dex promoted a reduction in the LAP2α nuclear amount, driving the AT condition towards a WT condition, but at the same time, increasing the LAP2α–Lamin A/C interaction.

Hence, dex was able to alter the above-mentioned picture, and it is likely that it also altered the downstream effectors. The role of dex in pRB phosphorylation in cell cycle regulation and apoptosis is well known^66–70^, but its role in interaction regulation has never been described. If dex, at 100 nM, is able to reduce the phosphorylation level of pRB^71^, the full LLRE complex (inhibitory on E2F1 target genes) should increase its quantity. On the contrary, based on our PLA results, we only observed an increase in Lamin A/C-E2F1 and LAP2α-Lamin A/C interactions, but a concomitant decrease in LaminA/C-pRB interactions and an unaltered interaction between pRB and E2F1. Similar results were obtained in both samples, although to a larger extent in AT samples.

It may be that the LLRE complex can also be constituted in other ways, either by the unbalanced partner mode, dissimilar to those described in the literature to date, or through dex, which may somehow alter its behavior. To link the LLRE complex composition to gene expression regulation we focused the analysis on two AT 648 hT differentially expressed gene promoters, shown to be E2F1 regulated by microarray bioinformatics output.

**Figure 12:**
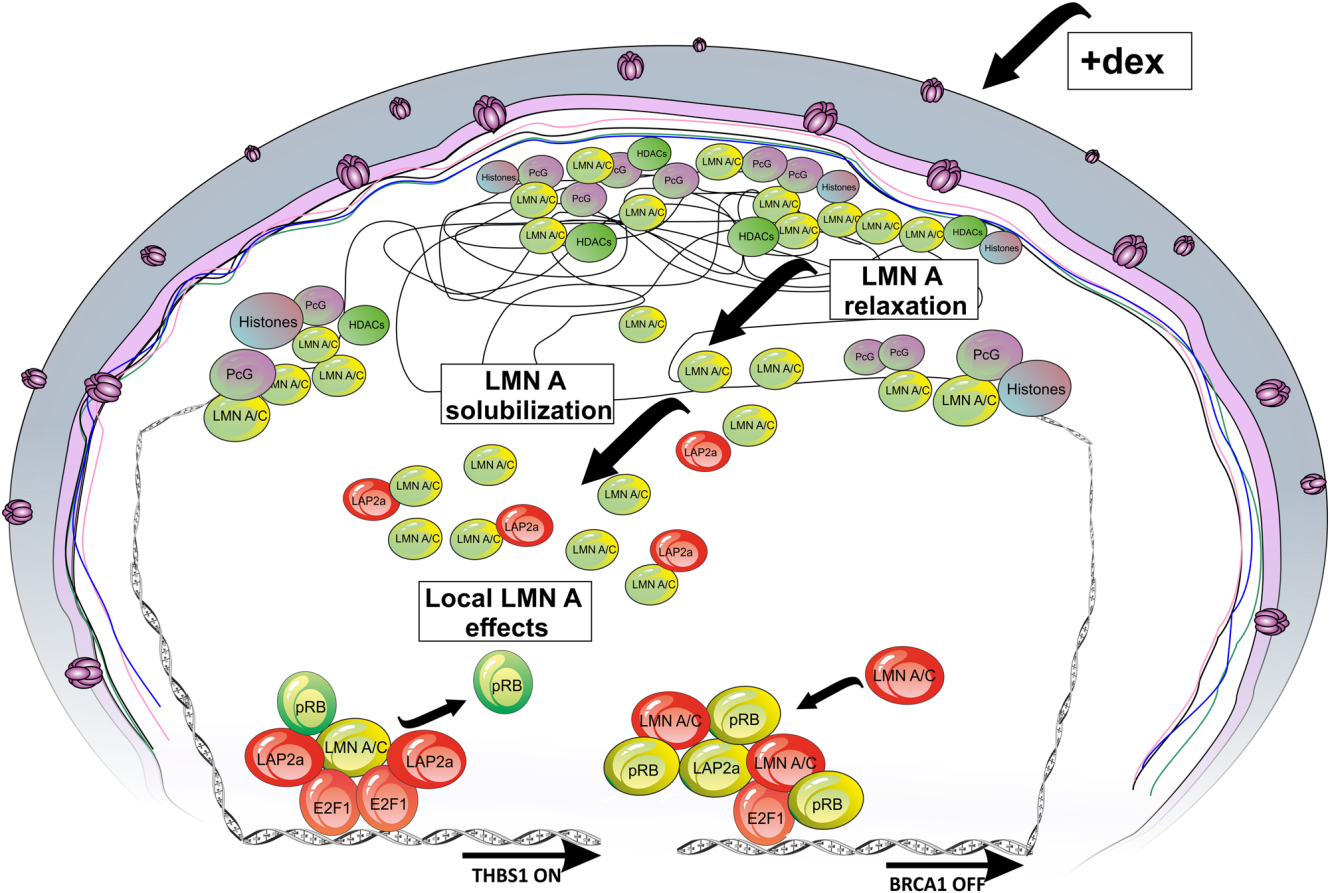
Condensing illustration of the effects of dex on Lamin A/C and downstream partners. In AT cells, globally dex promoted the release of Lamin A/C from higher level organized chromatin structures, as revealed by EDD peaks, probably by phosphorylation and LAP2α interaction. Locally, dex altered the LLRE composition determining a different outcome in gene regulation. The LLRE component colors on gene promoters represent increased (red), decreased (green) or unaltered (yellow) amounts of the indicated proteins on E2F1 binding site.

The downregulation of BRCA1 gene expression only seemed to depend on the increase in the Lamin A/C amount in the E2F1 promoter. The E2F1 transcription factor was physically present in all conditions and was likely inhibited by the Lamin A/C increase in the LLRE complex, the only difference compared to the WT sample, where BRCA1 expression is unaltered. The overexpression of THBS1 in AT 648 hT depended on the E2F1 increase in its promoter binding site together with a concomitant increase in LAP2α and a decrease in the pRB inhibitor. The possibility that LAP2α might directly bind E2F1, acting as an enhancer represents a future topic to be explored. It should be noted that the relationship between gene expression upregulation and an increase in LAP2α is consistent with the observation of Gesson et al.^43^, who noted that the A-type lamin is capable of binding euchromatin, and the latter is regulated by LAP2α. The proposed THBS1 and BRCA1 gene expression examples clearly did not consider all the other possible transcription factors and regulators that may play a role in their activation. An illustration summarizing the finding we describe in the paper is shown in Fig. 12. The present study has shown that dex treatment can somehow act on the nucleoplasm Lamin A/C dynamics, especially in AT cells, both on the high level of chromatin epigenetic structures and on the lower level of gene regulation by driving differential interaction with E2F1 partners. In the last few years also some muscular dystrophies caused by Lamin A/C mutations have been treated with corticosteroids^72^, thus it may be useful to enhance our knowledge of how this drug works in laminopathies, pathologies that share some characteristics with AT, and in other conditions, including progeroid syndromes and aging were Lamin A/C can have a role.

## Methods

### Cell culture and treatments

Primary fibroblasts WT AG09429 (ATM + / +), AT GM00648 (ATM -/-), AT GM02052 (ATM -/-) were obtained from Coriell Institute (Camden, NJ, USA). The hTERT immortalized cells WT AG09429 hTERT (WT hT) and AT GM00648 hTERT (AT 648 hT) were obtained as described previously^47^.

Primary and hTERT fibroblasts were grown in MEM (Eagle formulation). The medium was supplemented with 2 mmol/l L-glutamine, 100 U/mL penicillin and 0.1 mg/mL streptomycin (Sigma Aldrich), with the addition of 15% fetal bovine serum (Thermo Fisher Scientific) for primary cells, and of 10% fetal bovine serum (Thermo Fisher Scientific) and 10 mM glucose for hTERT cells.

All cells were incubated at 37 °C with 5% CO2 and treated with 100 nM dex for 72 h prior to each analysis. Dimethylsulfoxide (DMSO) was used as the drug vehicle and thus was administered in untreated cells as a control.

### Antibodies

The antibodies listed below were purchased from commercial sources: anti Lamin A/C (Cell Signaling Technology CST #4777 and Diatheva #ANT0072), FITC-conjugated anti Lamin A/C (CST #8617), anti LAP2α (BETHYL, A304-839A-M), anti phospho S22 Lamin A/C (CST #13448), anti phospho S404 Lamin A/C was kindly provided by Prof. Marmiroli^42^, anti pRB (CST #9309, BETHYL #A302-433A-T), anti E2F1 (Santa Cruz Biotechnology #sc-251, BETHYL #A300-766A-M). HRP-conjugated secondary antibodies used for western blotting were purchased from Bio-Rad, while fluorescence-conjugated secondary antibodies were purchased from MERCK.

### Western blotting

Total proteins were extracted by using the Protein Extraction Reagent Type 4 (P4, MERCK). Cells were sonicated with 10 pulses of 15 s at 45 Watts Labsonic 1510 Sonicator (Braun) and clarified by centrifugation for 10 min at 10,000 RCF.

Nuclear fractions were obtained lysing the cells in Buffer A (10 mM Hepes/KOH pH 7.9, 1.5 mM MgCl2, 10 mM KCl, 2 mM dithiothreitol (DTT), 0.1% Nonidet-P40) containing protease inhibitors (Roche Applied Science) and phosphatase inhibitors (10 mM NaF, 2 mM Na_3_VO_4_) on ice for 10 min. Cells were centrifuged at 5000 RCF for 10 min and the supernatants containing the cytosolic fraction were discarded. The nuclear pellets were then lysed as described by Marullo et al.^50^. After clarification, the supernatants containing the nuclear fractions were collected. Protein concentration was determined by Bio-Rad Protein Assay. Twenty micrograms of proteins were separated by SDS–PAGE according to the Laemmli protocol (Novex 10–20% Tris–Glycine gels) and then transferred to nitrocellulose (0.22 µm, Bio-Rad) by wet transfer and Towbin blotting buffer (50 mM Tris, 150 mM NaCl, 20% (v/v) methanol). Membranes were probed with the primary antibodies and corresponding secondary HRP-coupled antibodies diluted in 5% w/v non-fat dry milk or 5% BSA in TBS 0.1%Tween. Immunoreactive signals were acquired using the enhanced chemiluminescence (Advansta) by film or acquired by ChemiDoc Touch Imaging System (Bio-Rad). The whole lane normalization strategy was adopted in all western blot analyses by using a trihalo-compound for protein visualization^73,74^. Image analyses were performed by Image-Lab v6 (Bio-Rad). For Lamin A/C solubilization test in hTERT cells, nuclei obtained after buffer A incubation were subsequently treated with detergent containing buffer (NP40 0.5%) as described by Kolb et al.^29^ and after centrifugation, the pellets were resuspended in Protein Extraction Reagent Type 4. For western blotting, a ratio of 3/1 of pellets soluble fractions were loaded on gels. Immunoreactive signals were acquired as previously described. Full-length images are included in Supplementary Material. The missing of adequate length of some original images is due to blots cut prior to hybridization with antibodies, or because maximum zoom during signal acquisition by ChemiDoc Touch Imaging System was employed.

### Indirect immunofluorescence microscopy

IF was performed as previously reported^47^. Briefly, the cells to be examined were seeded in Lab-Tek II chamber slides (NUNC). After stimulation, they were fixed for 10 min with 4% formaldehyde and then with 100% cold methanol. They were subsequently permeabilized with 0.5% NP-40 in PBS for 10 min. After performing the blocking procedure for 1.5 h at room temperature, primary antibodies were applied in 0.1% Triton X100, 1%BSA in PBS overnight at 4 °C.

After three washes by 0.1% Triton X100 in PBS at room temperature, the cells were incubated with secondary anti-mouse or anti-rabbit FITC-conjugated antibody (Sigma-Aldrich) in 0.1%Triton X100, 1%BSA in PBS for 1 h at 37 °C. After washing procedures, the slides were mounted and embedded with ProLong Antifade (Thermo Fisher Scientific) and observed by Olympus IX51.The images were acquired by the ToupCam camera (ToupTek Europe) or by the Leica TCS SP5 confocal setup mounted on a Leica DMI6000 CS inverted microscope (Leica Microsystems). Image analyses were performed by ImageJ (NIH).

### PLA experiments

Protein interaction detection was performed using the Duolink system (Sigma-Aldrich) according to the manufacturer’s instructions. Cells were seeded and fixed as previously described. The antibody specificity setup was determined by examining the experimental control outputs at different antibody dilutions. Once each optimum condition was found, the PLA experiments were carried out. Nuclei were stained with 4′,6-diamidino-2-phenylindole (DAPI) at a final concentration of 0.2 µg/ml or were highlighted by FITC anti Lamin A/C adopting the approach indicated by the manufacturer Duolink. Signals were anlyzed by ImageJ using nuclear ROI and subtracting the average background.

### Chromatin immunoprecipitation followed by deep sequencing (ChIP-seq)

ChIP was performed for each culture condition. Briefly, cells were cross-linked for 10 min with 1% formaldehyde and the nuclei were prepared by cell lysis buffer (5 mM HEPES–KOH pH7.5, 85 mM KCl, 0.5%NP-40 1 × complete protease inhibitor, 10 min. on ice). Nuclei-containing pellets were resuspended in lysis buffer (50 mM Tris–HCl pH8, 10 mM EDTA, 1%SDS, 1 × complete protease inhibitor) and subsequently sonicated by Bioruptor Plus for 12–16 cycles in order to obtain a comparable fragment size range among the samples, between 100 and 600 bp. Fifty micrograms of input chromatin was diluted in binding buffer (Final: 0.2% SDS, 1%Triton X100, 150 mM NaCl, 2 mM EDTA, 0.5 mM EGTA, 10 mM Tris pH8.5 1 × complete protease inhibitor) and incubated with antibodies overnight after a pre-cleaning step. Complexes were purified with A/G beads and after washing, chromatin was de-crosslinked, RNAase A and proteinase K were treated, and DNA was purified. The ChIP was performed in triplicate.

The libraries to be processed by Illumina cBot were prepared by ovation Ultralow Library System v2 kit (NuGEN, San Carlos, CA) following, for each step of the procedure, the manufacturer’s instructions. After clusters generation on the flow cell, single-end 50 bp mode sequencing was utilized in HiSeq2500 (Illumina, San Diego, CA). The CASAVA 1.8.2 version of the Illumina pipeline was used to process raw data for both format conversion and de-multiplexing.

After GC correction, regions of enrichment (peaks) were identified using EDD version 1.0.2 compiled by Python v2.7 on Xfce Desktop Environment v4.8. The parameters were set “-b 10 –g 5 –FDR 0.1” (bin size 10 kb, gap penalty 5). ChIP-seq data (peaks and log ratios) were visualized with the Integrative Genomics Viewer^75^, while genome interval manipulations were performed by BedTools.

### Microarrays

Total RNA extracts were obtained from all tested cell lines using the RNeasy Plus Mini Kit (QIAGEN). RNA labelling was performed using the WT Pico kit and Clariom D chips were used and subsequently imaged by the GeneChip Scanner 3000 7G (Affymetrix). The data analyses, after pre-processing at the probe level (CEL files), were performed by RMA background adjustment, quantile method for normalization and median polish for summarization. Differentially expressed genes were selected by Affymetrix TAC console 4, employing a linear F/C > and < 1.5 and FDR < 0.05 between three technical replicates of control and treated samples. Network analysis was performed by the Reactome FI Functional Interaction Network plugin for Cytoscape^76,77^. The matching and retrieving of genomic intervals of up- or downregulated genes with genomic regions (obtained by EDD) were performed by list comparison software developed in-house. The probability of transcription factors activating genes selected by microarray analyses was computed by TFactS^78,79^ using the Q-value and FDR to 0.05.

### Chromatin immunoprecipitation followed by qPCR

For THBS and BRCA1 promoter- pRB, LAP2α, E2F1 and Lamin A/C binding studies, the chromatin was prepared as previously described and immunoprecipitated with the specific antibodies. MOCK samples were prepared as controls. The obtained purified DNAs, recovered as previously described, were amplified by qPCR using the SYBR Green Premix Ex Taq Tli RNaseH Plus (Takara). The employed primers surrounding the E2F1 binding site in the BRCA1 promoter^80^ were: forward 5′-CACAGGTCTCCAATCTATCCA-3′ and reverse 5′-GTCAGAATCGCTACCTATTGTC-3′, while for THBS1^81^ they were: forward 5′-TTTCTAGCTGGAAAGTTGCG-3′ and reverse 5′-GTAGAGGTTGCTCCTGGAGAG-3′. The qPCRs efficiency was established by standard curves (on average 95% efficiency), ensuring that last standard dilution was sample CT inclusive. Amplification plots were analyzed using the ABI PRISM 7500 sequence detection system (Applied Biosystems) and the relative DNA amounts were calculated by the ½ ^Ct^ method.

### Statistical analysis

GraphPad Prism was used for statistical analyses and graph generation. Statistical tests were chosen according to sample size and variance homogeneity. The following tests were used: t-test, Welch Test, test U Mann–Whitney, Wilcoxon test. Means or medians were considered statistically different with *p* ≤ 0.05.

## Supplementary information

Supplementary Information 1.

Supplementary Information 2.

Supplementary Information 3.

Supplementary figures.

## Data Availability

All data generated or analyzed during this study are included in this published article (and its Supplementary Information files) and available from the corresponding author on reasonable request. Microarray raw data are under investigation for splicing analysis at the time of paper submission.
