# Supplementary material for: The nucleoplasmic interactions among Lamin A/C-pRB-LAP2α-E2F1 are modulated by dexamethasone
Source: Sci Rep. 2021 May 12;11:10099. doi: 10.1038/s41598-021-89608-3 (PMC8115688; doi:10.1038/s41598-021-89608-3)

Supplementary figure S1

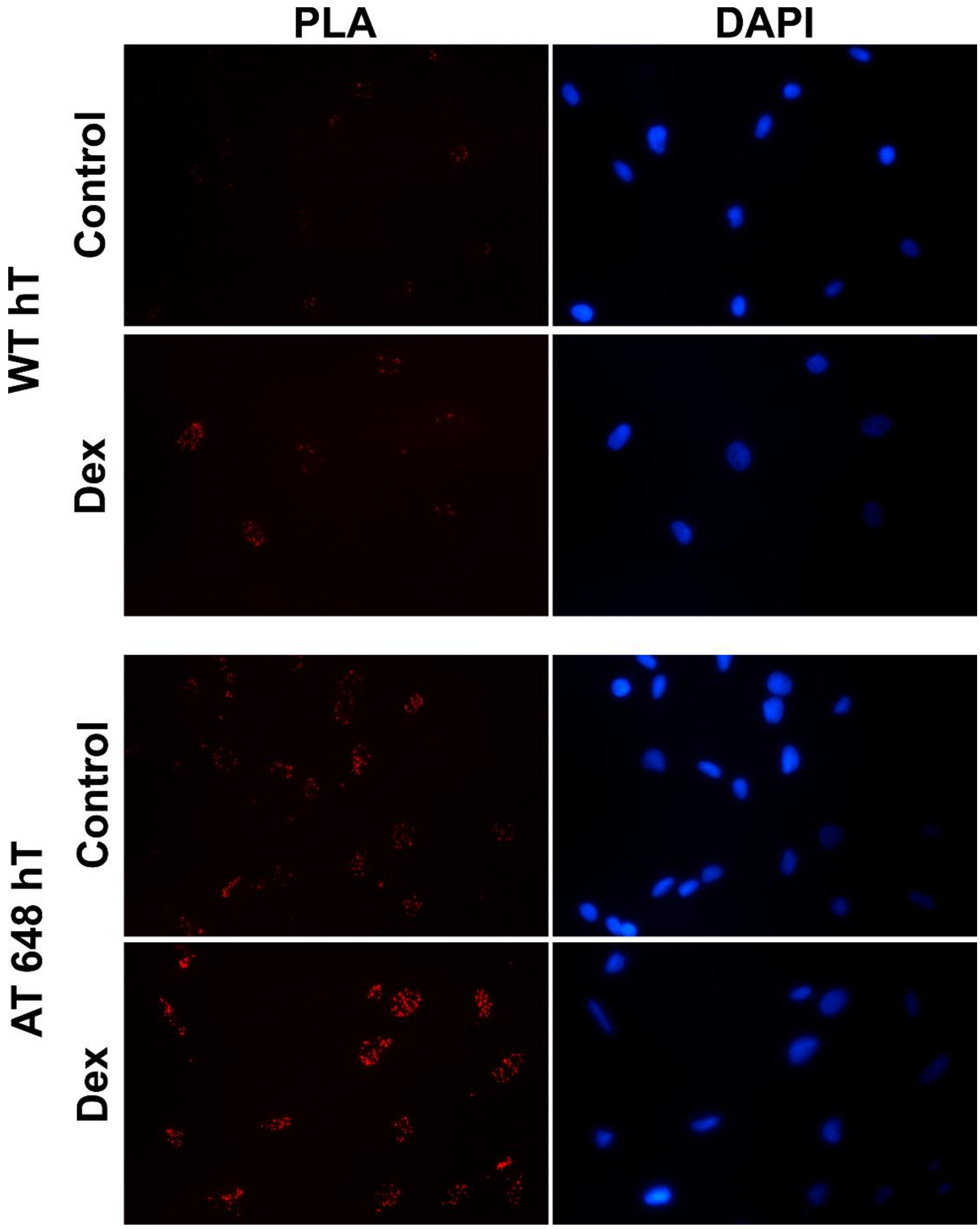

Supplementary figure S2

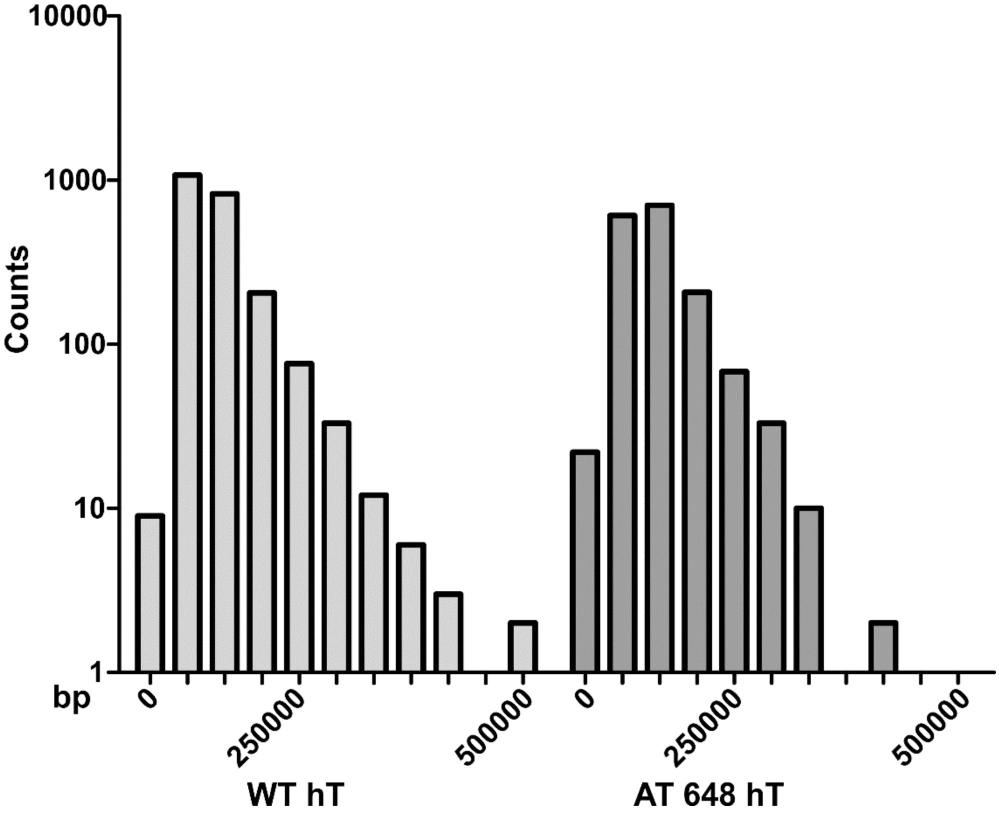

Supplementary figure S3

WT hT

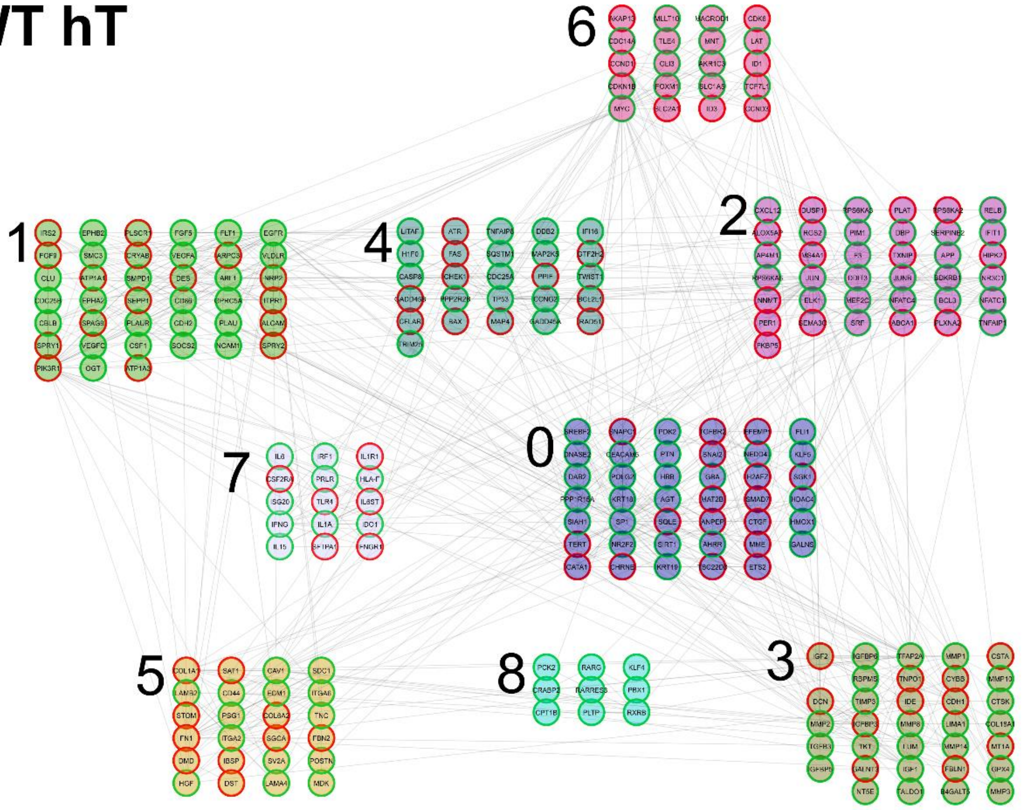

AT 648 hT

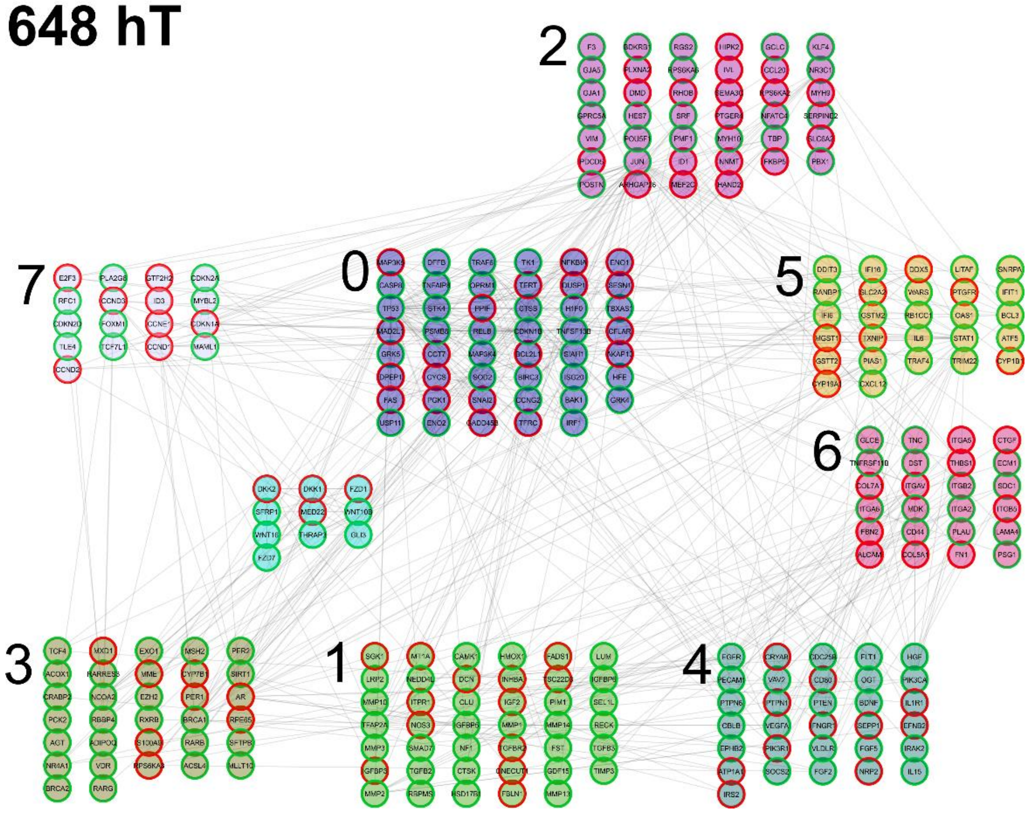

Supplementary figure S4

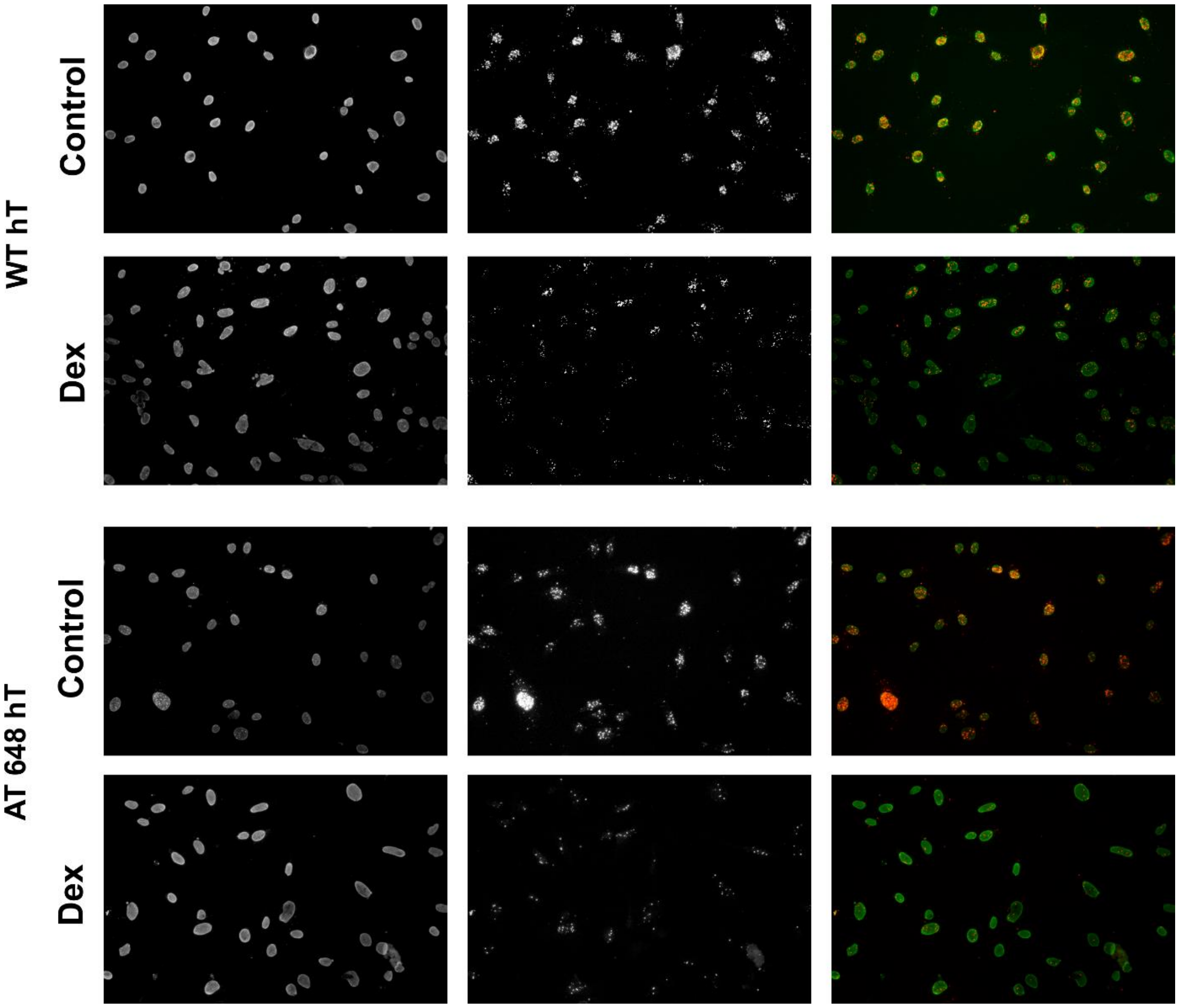

Supplementary figure S5

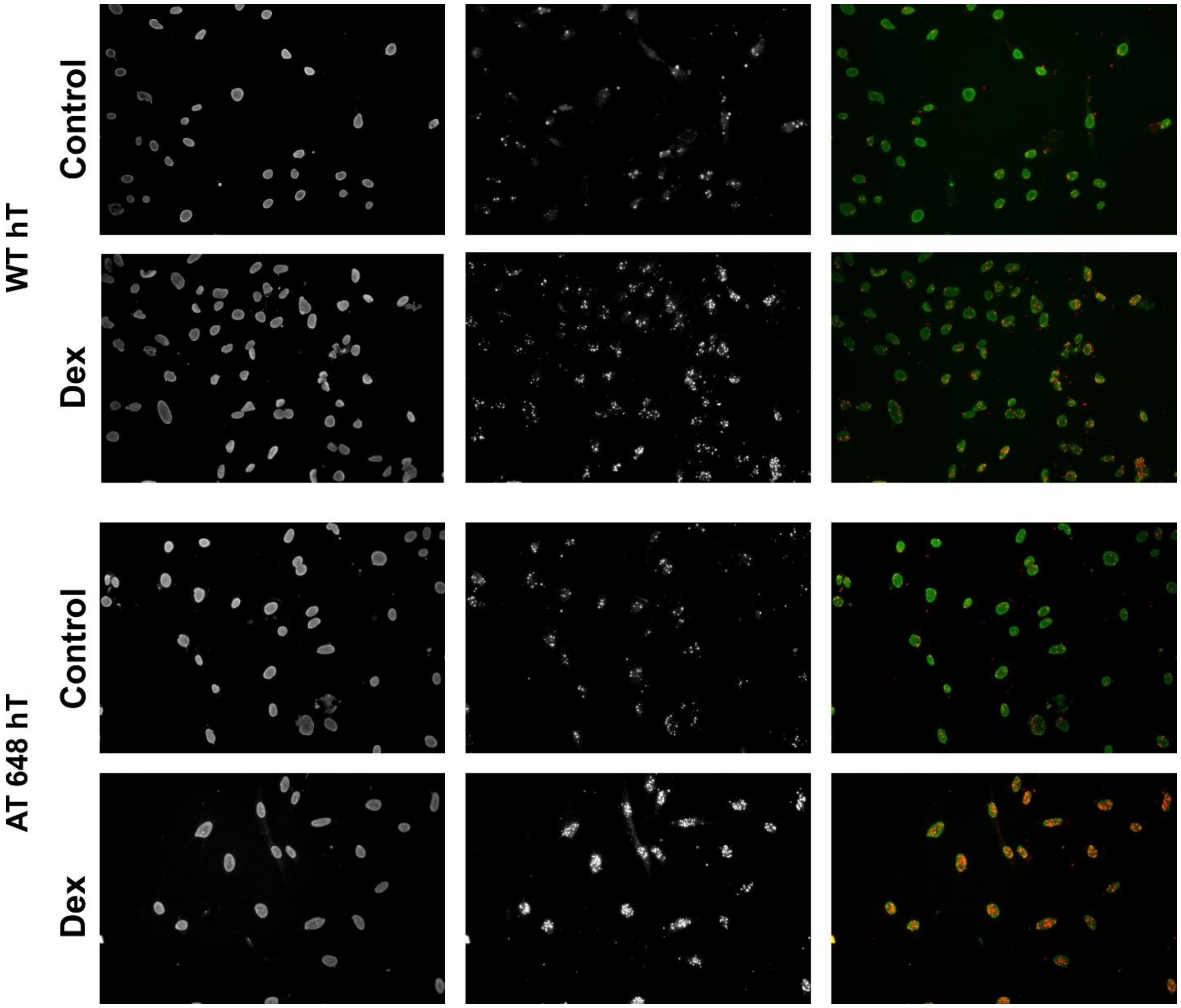

Supplementary figure S6.

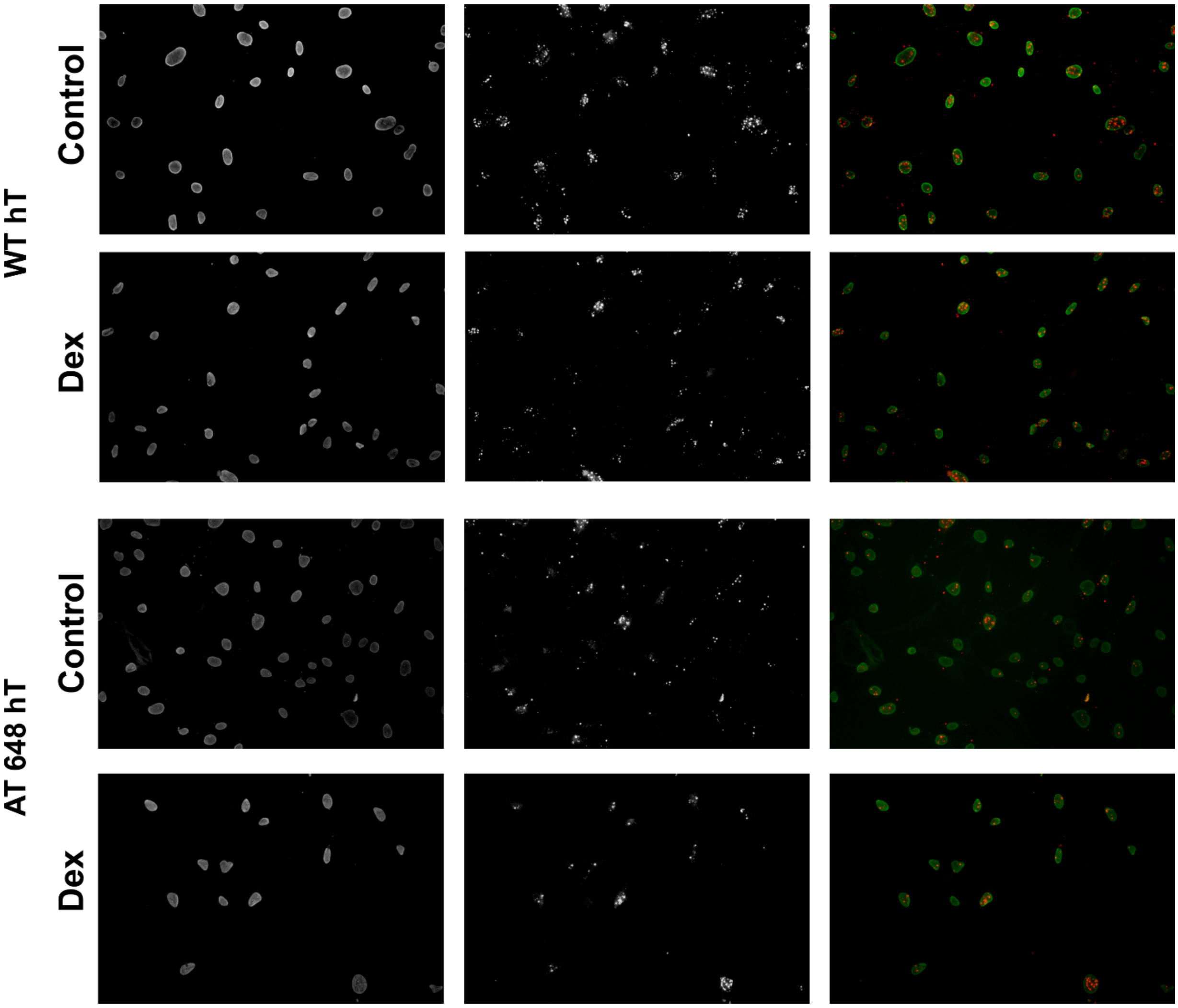

S1O

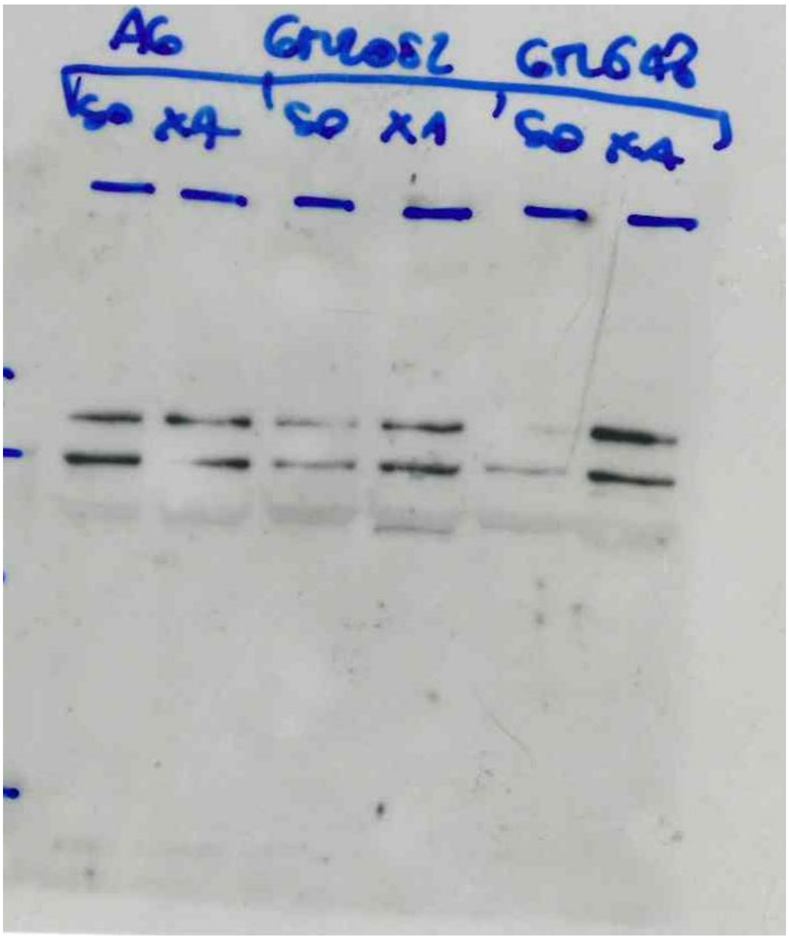

S3O_1

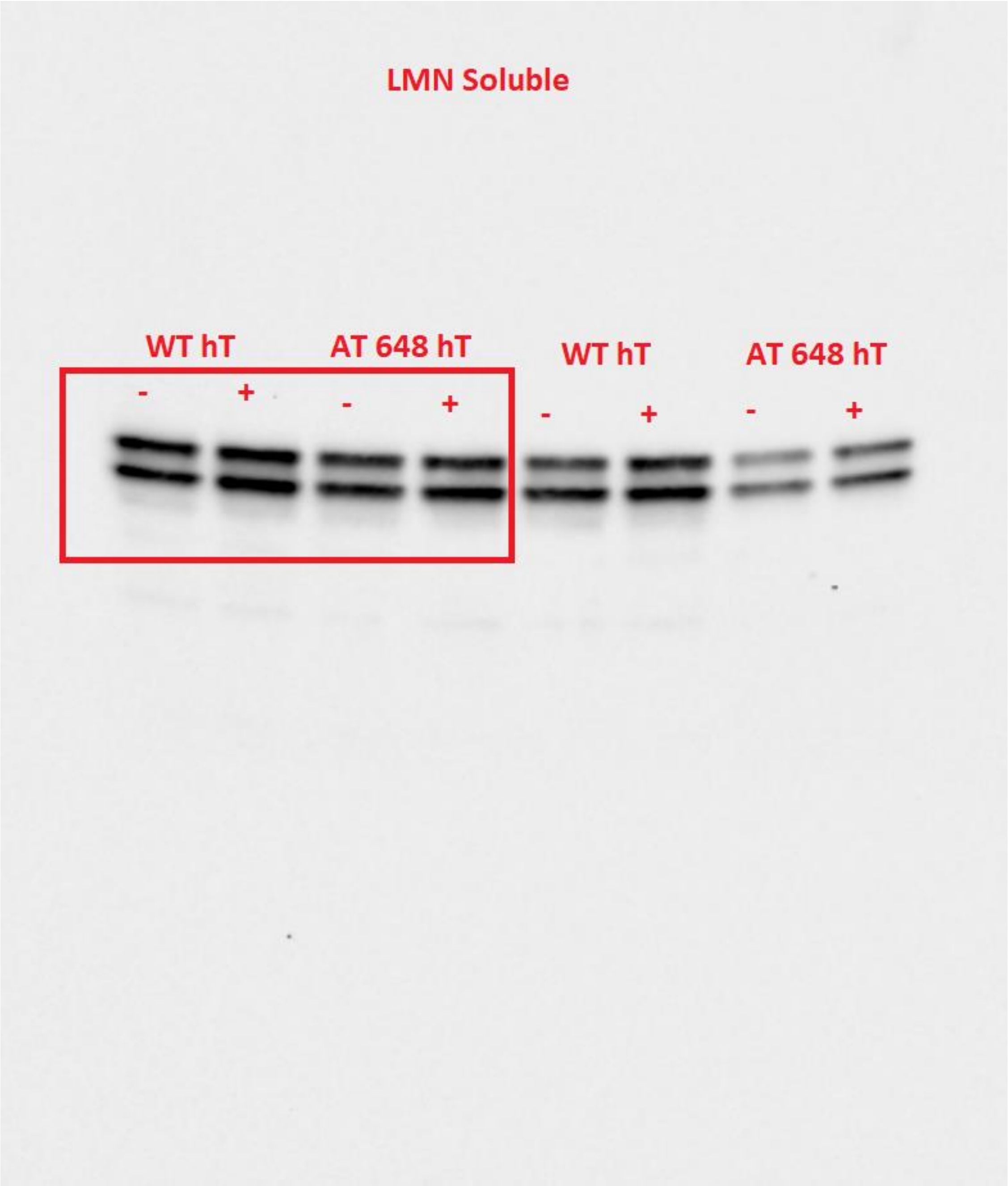

S3O_2

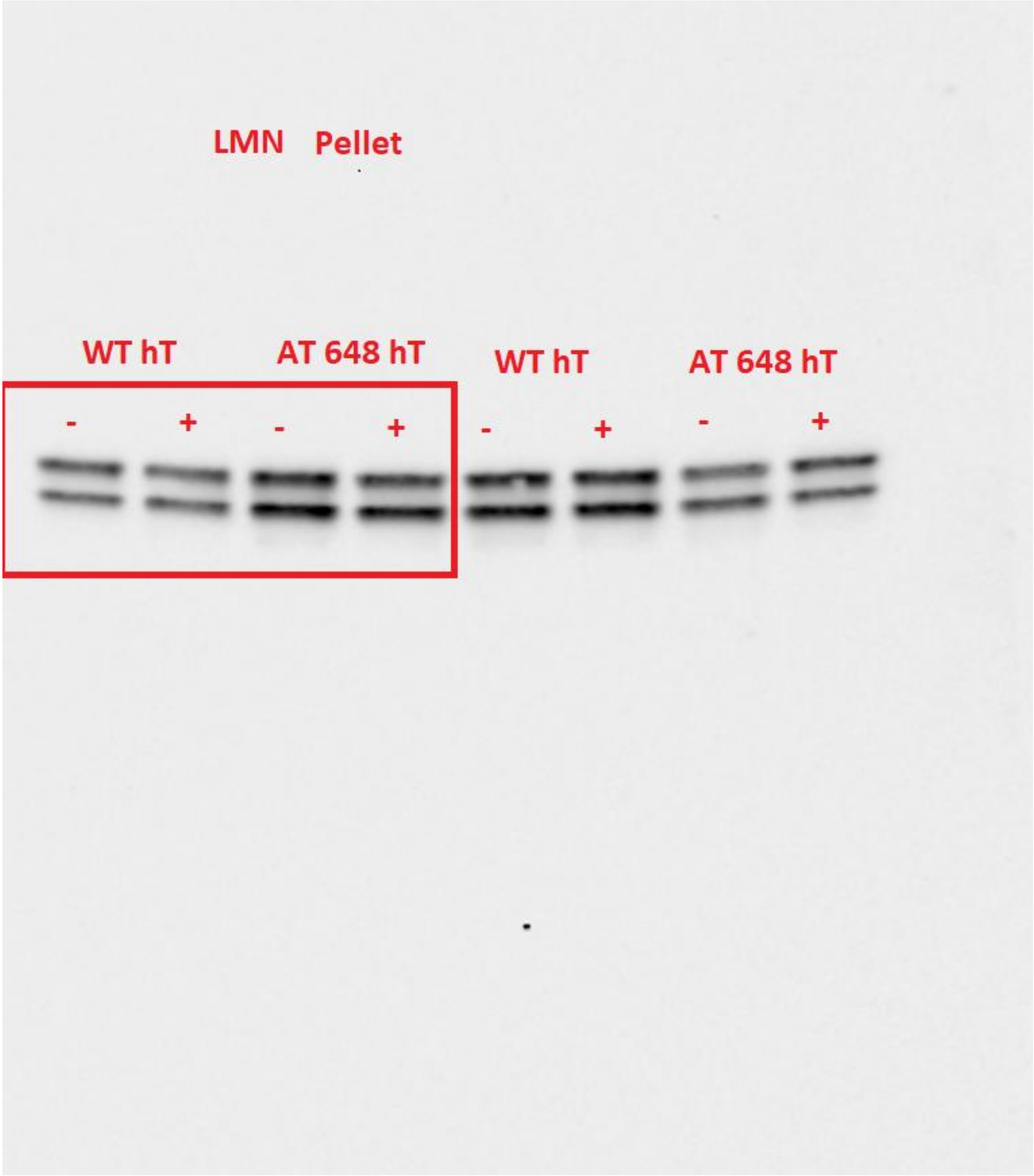

S3O3_3

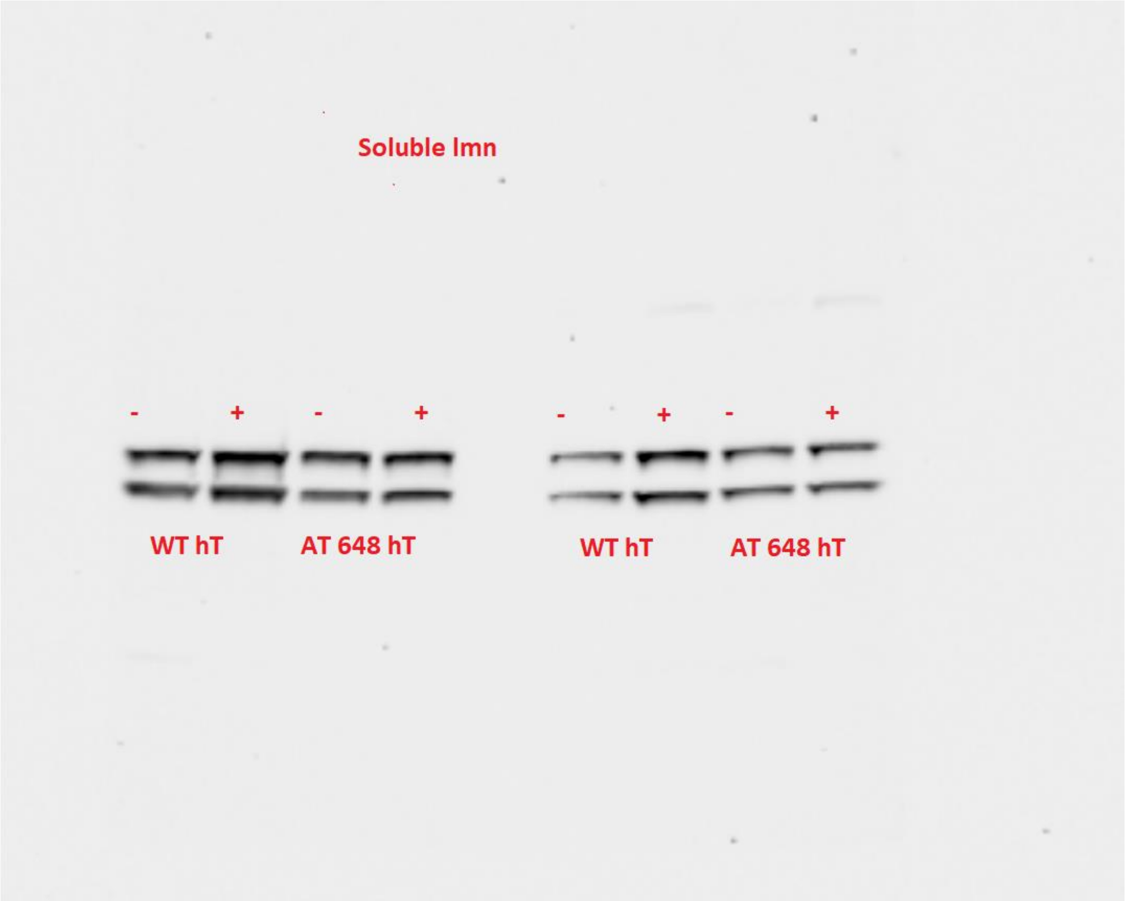

S3O_4

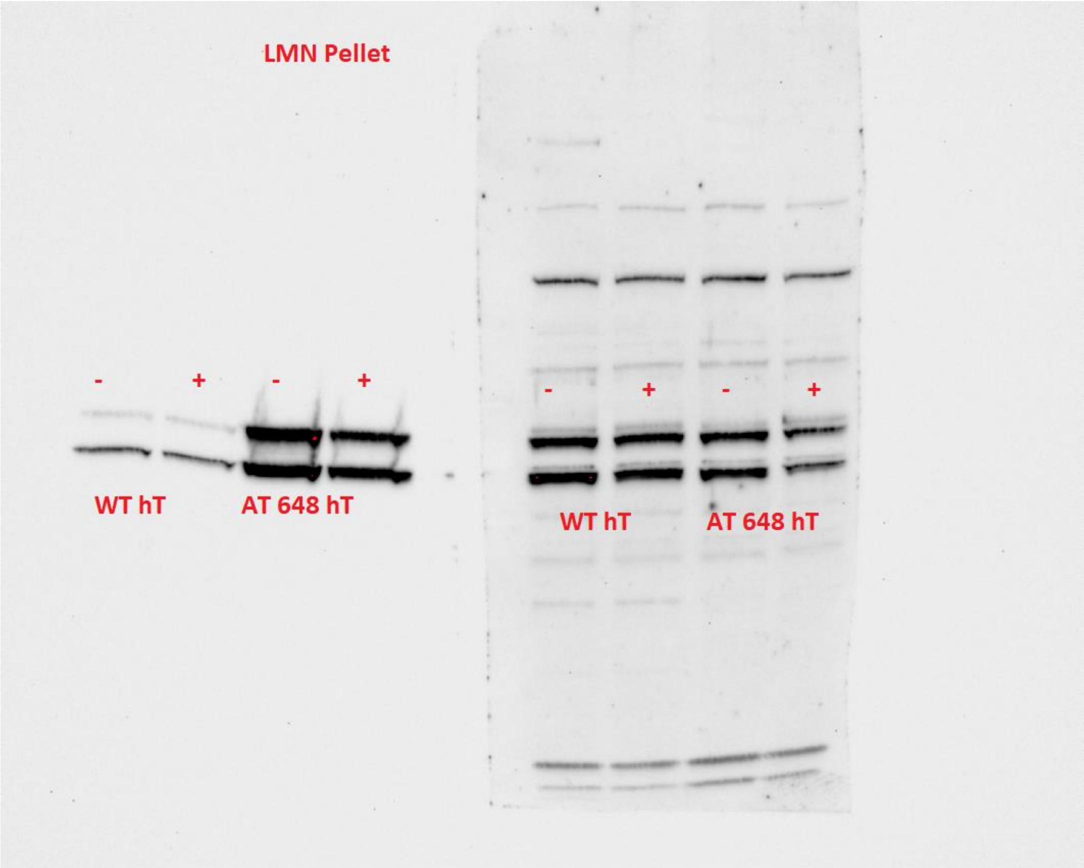

S6O a1

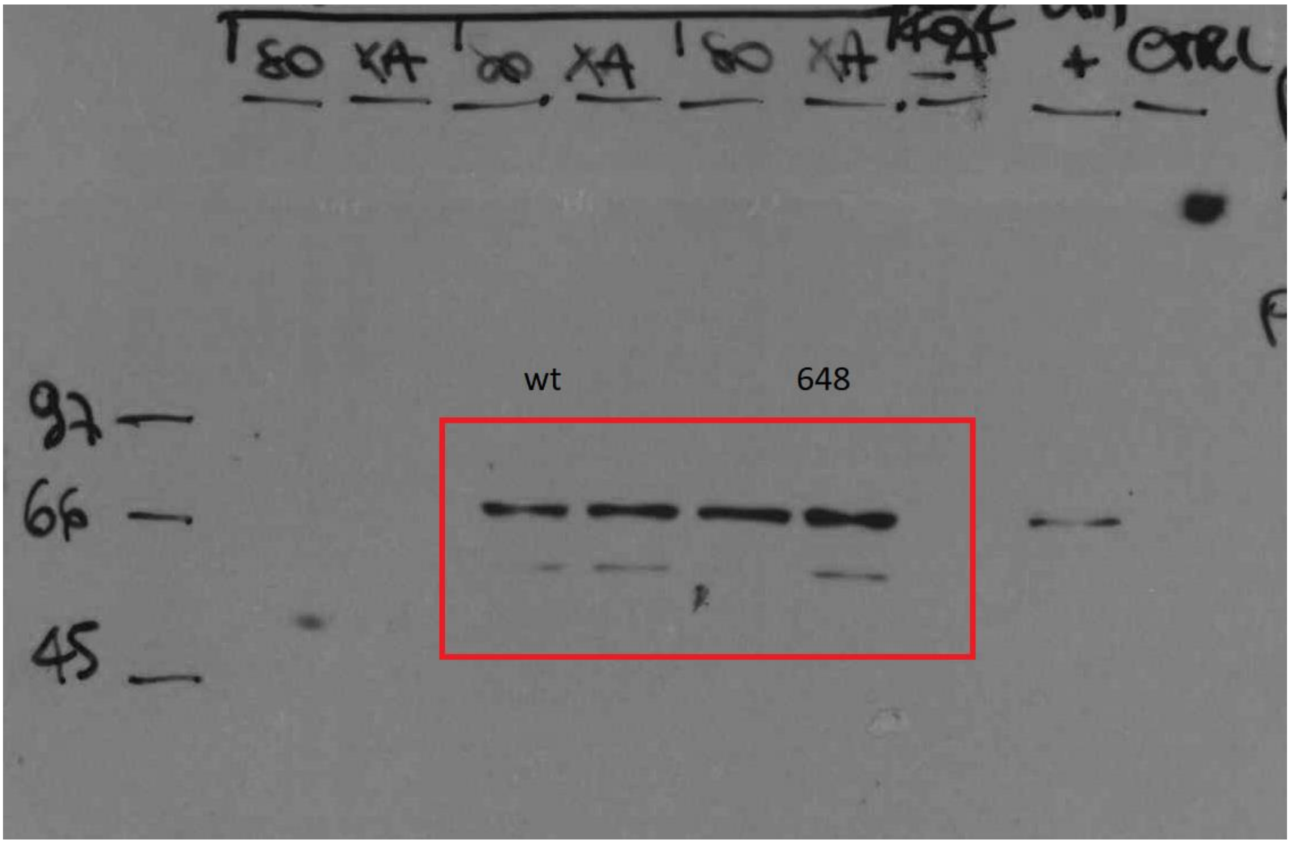

S6O a2

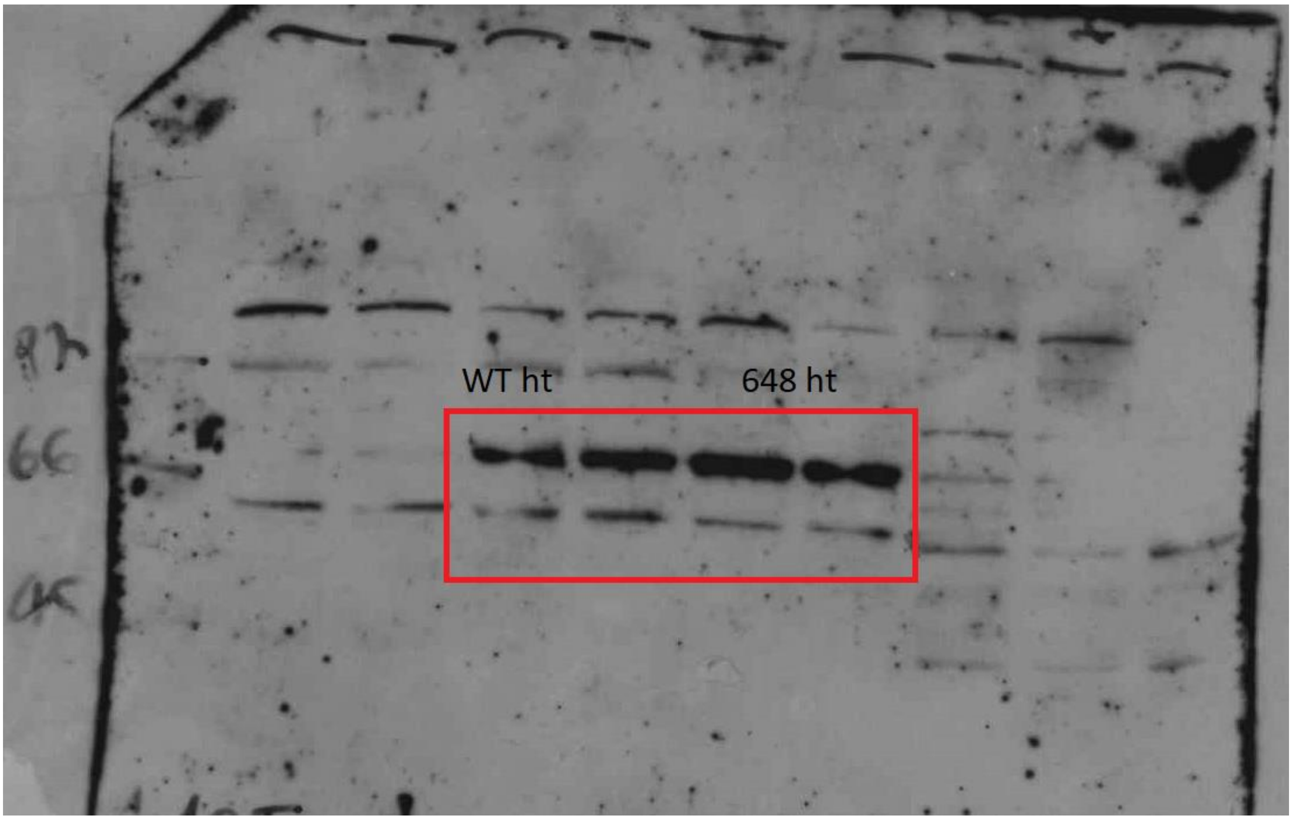

S6O b1

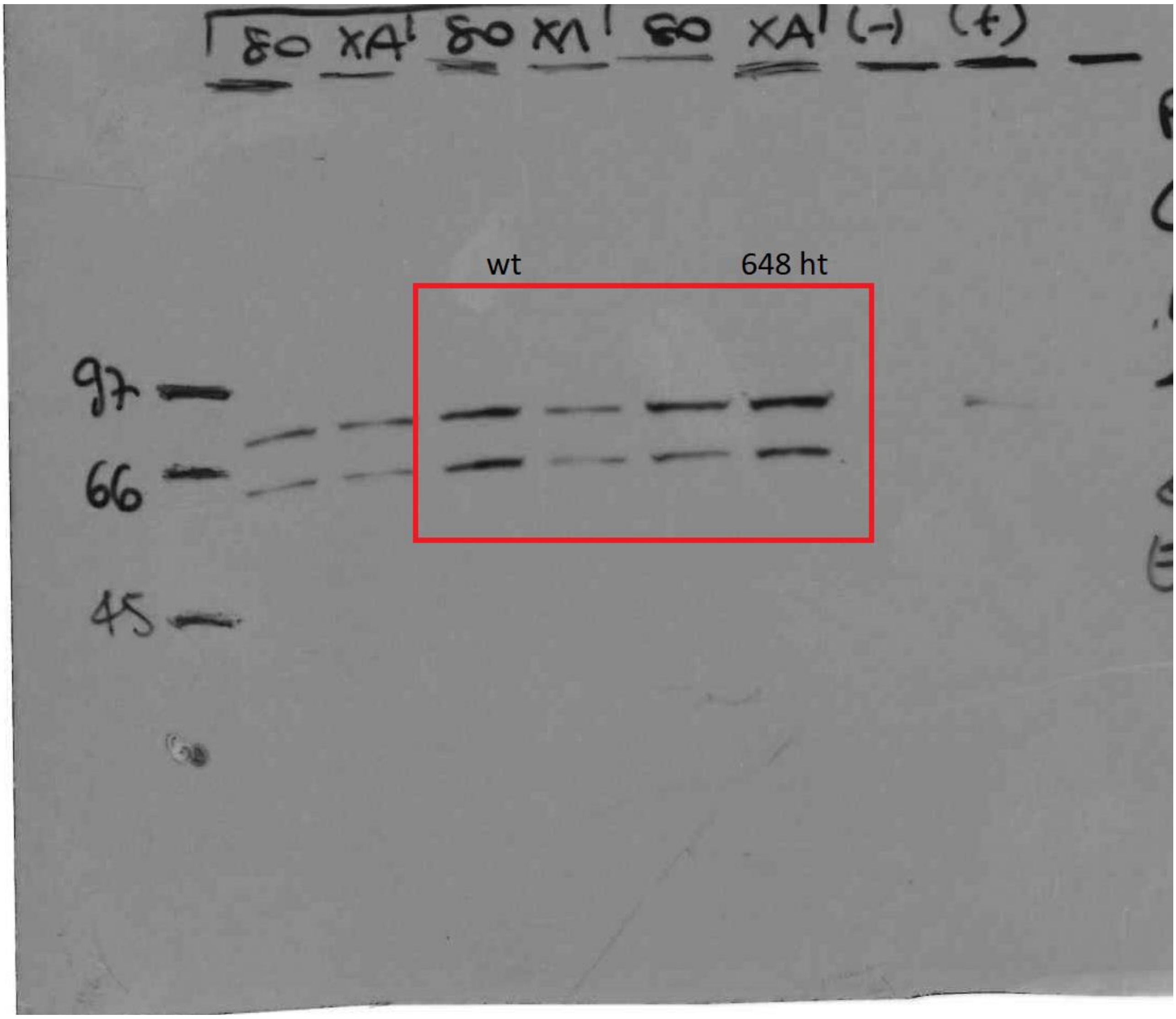

S6O b2

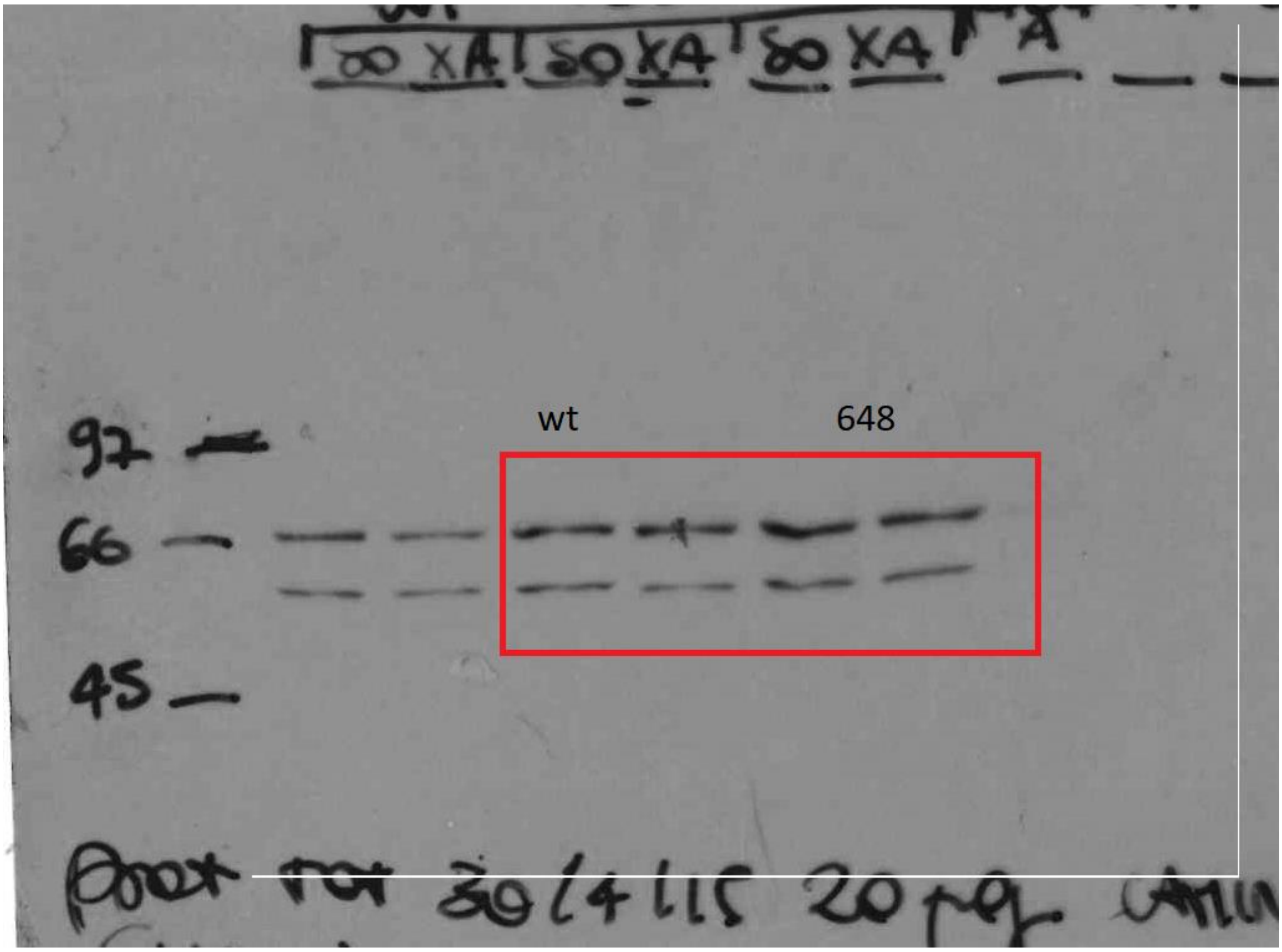

S6Oa1_648

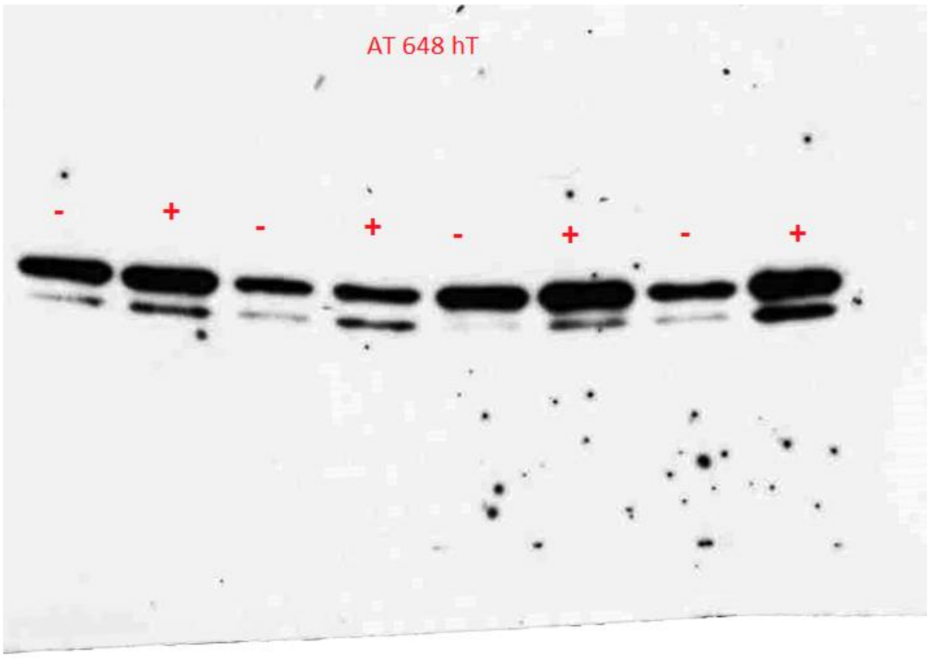

S6Oa1_WT

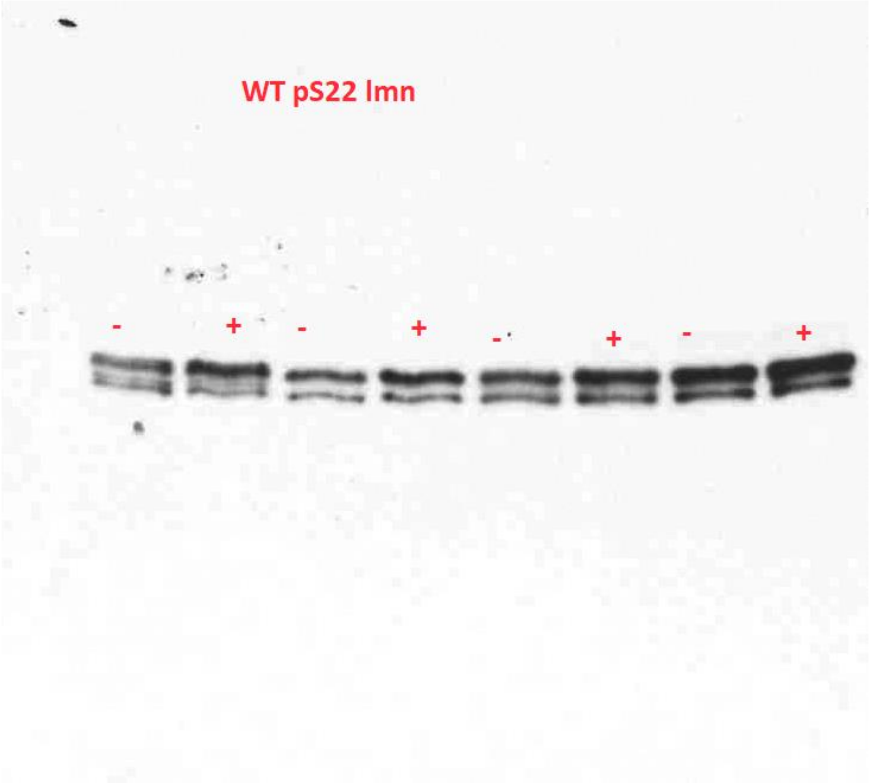

S6Oa2_648

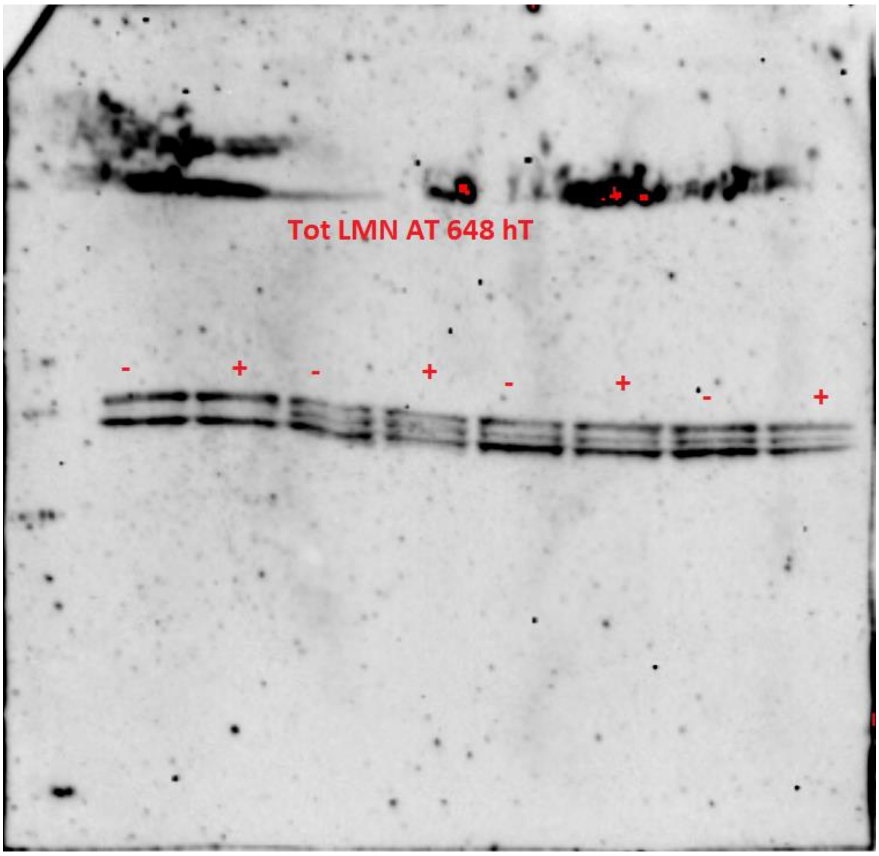

S6Oa2_WT

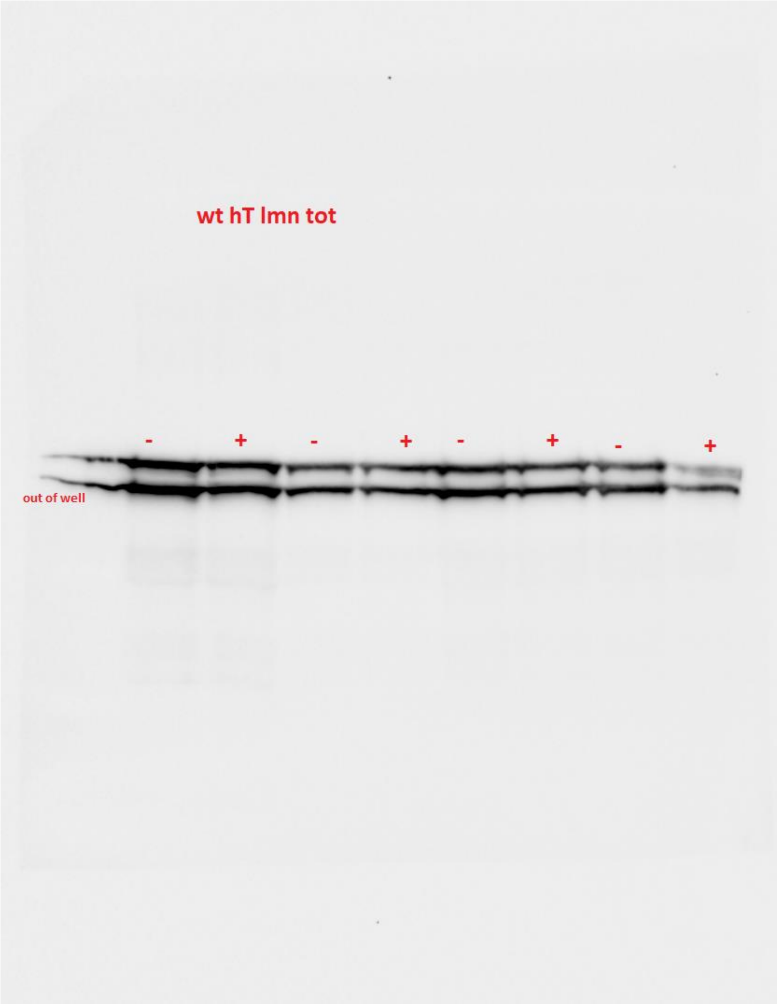

S6Ob1_648

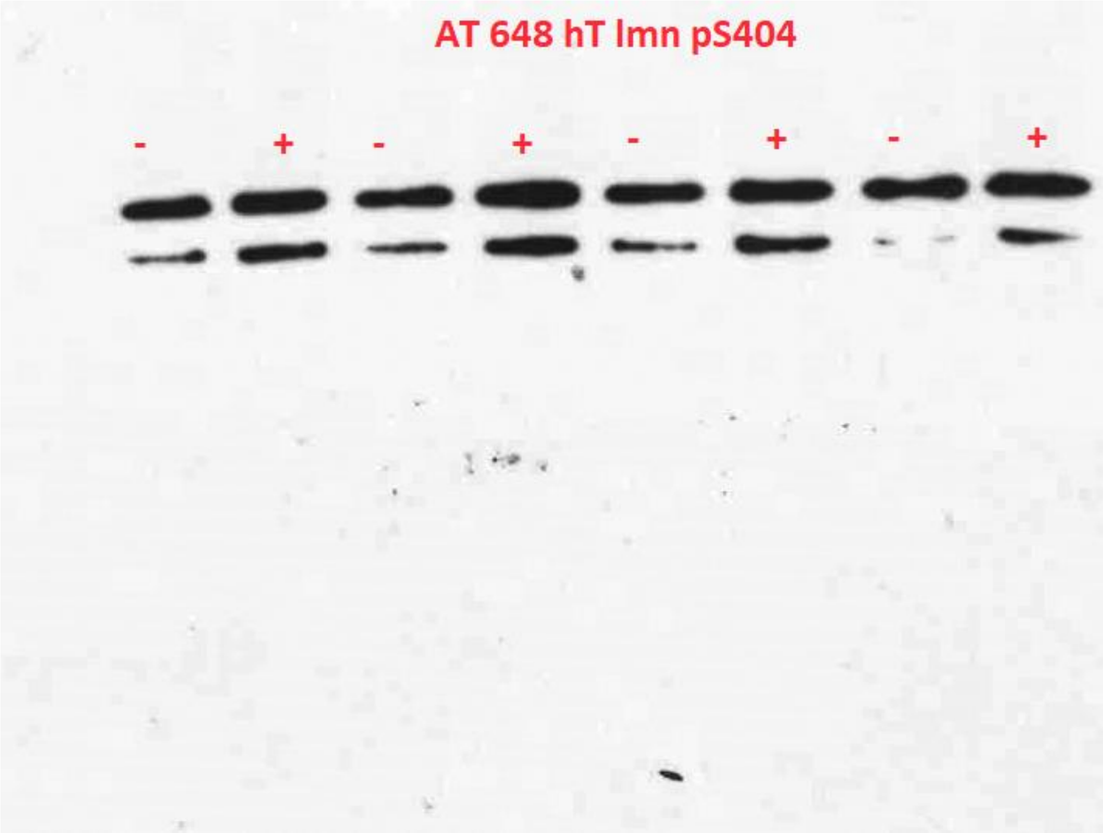

S6Ob1_WT

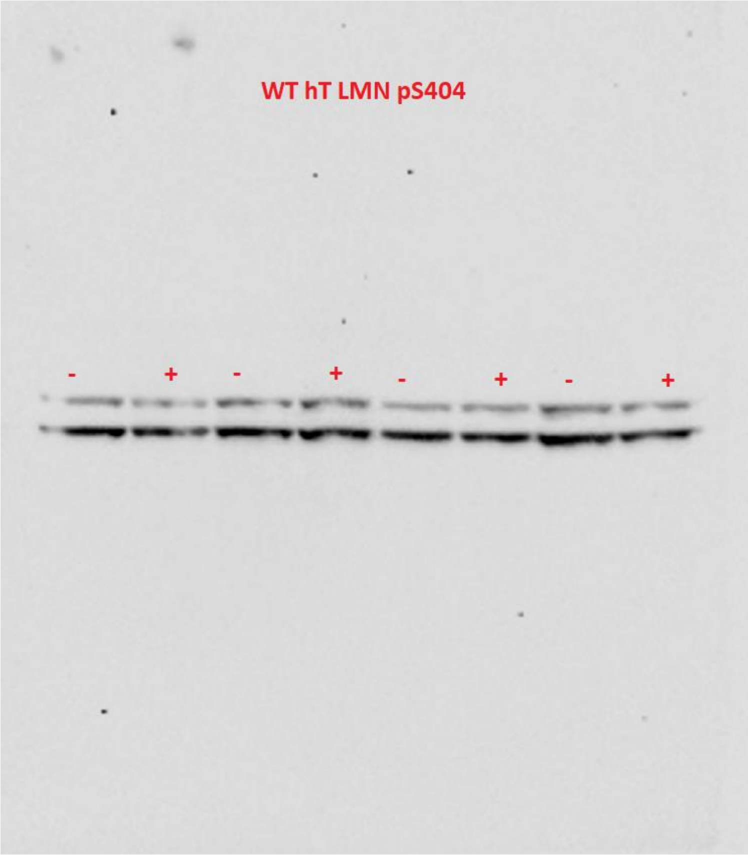

S6Ob2_648

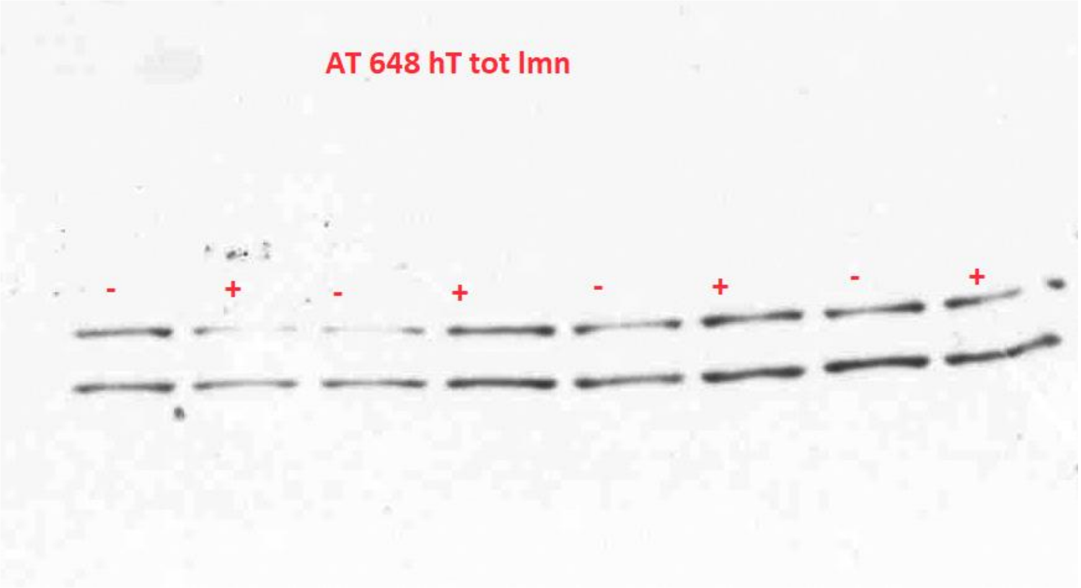

S6Ob2_WT

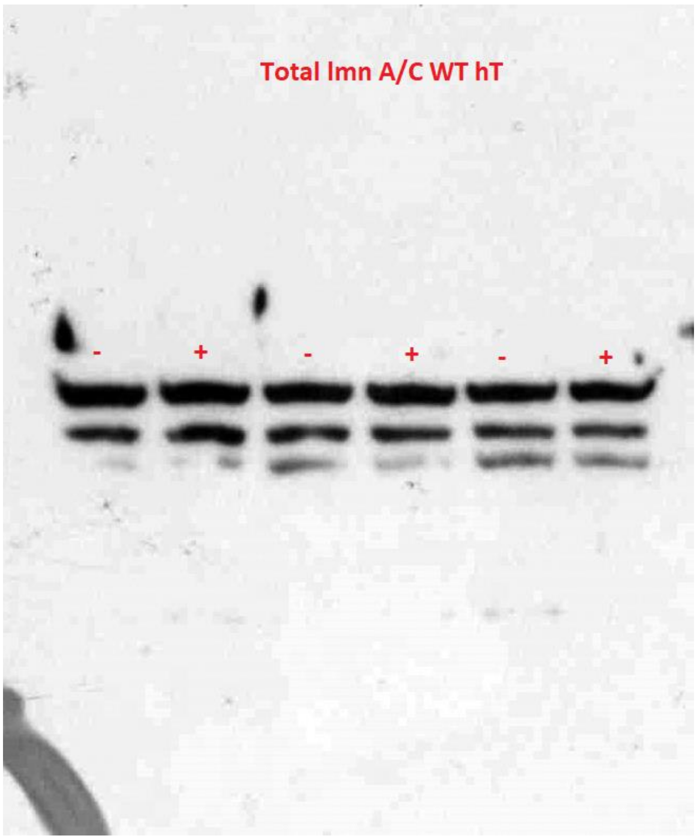

S8O a1

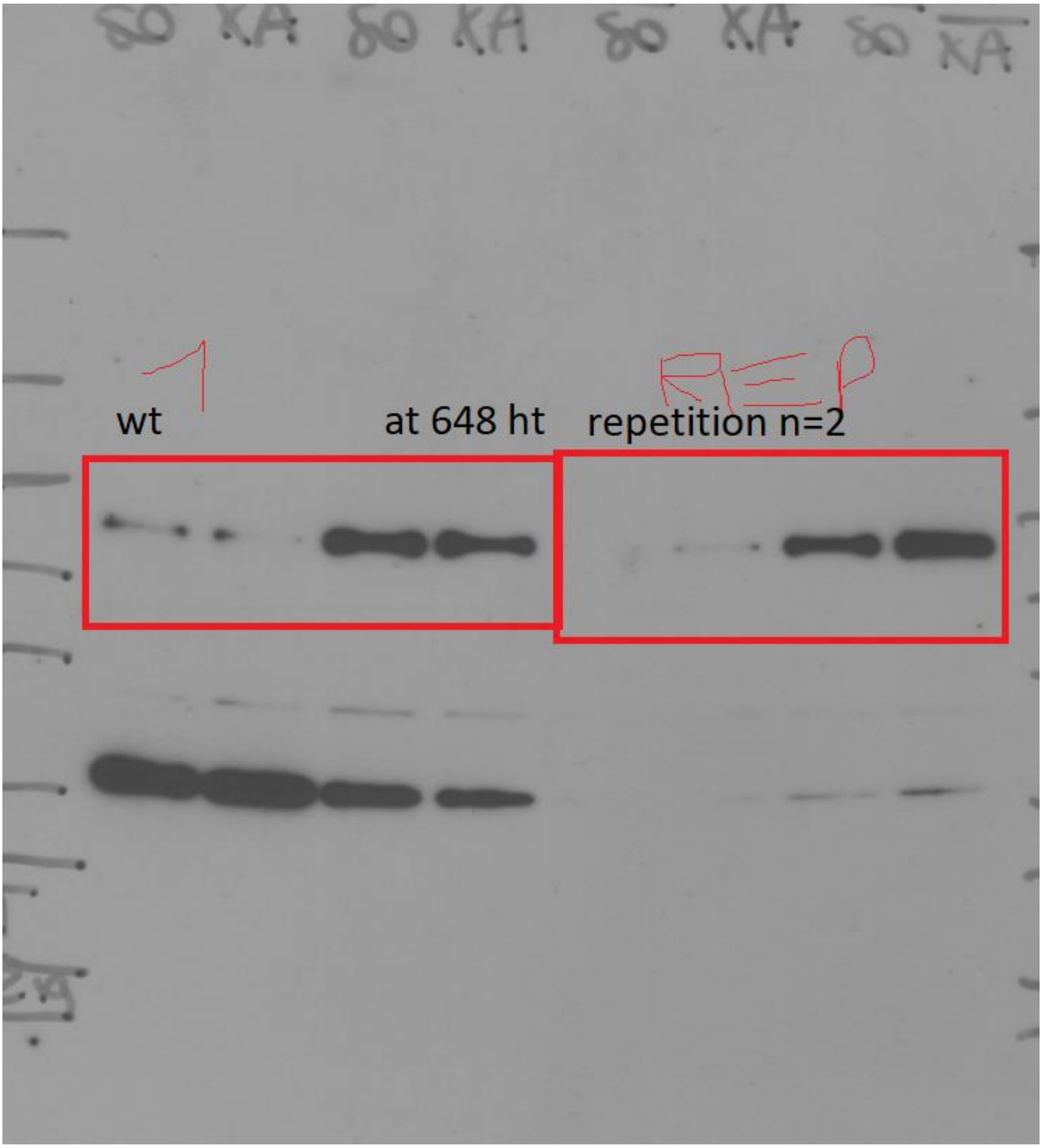

S8O a3

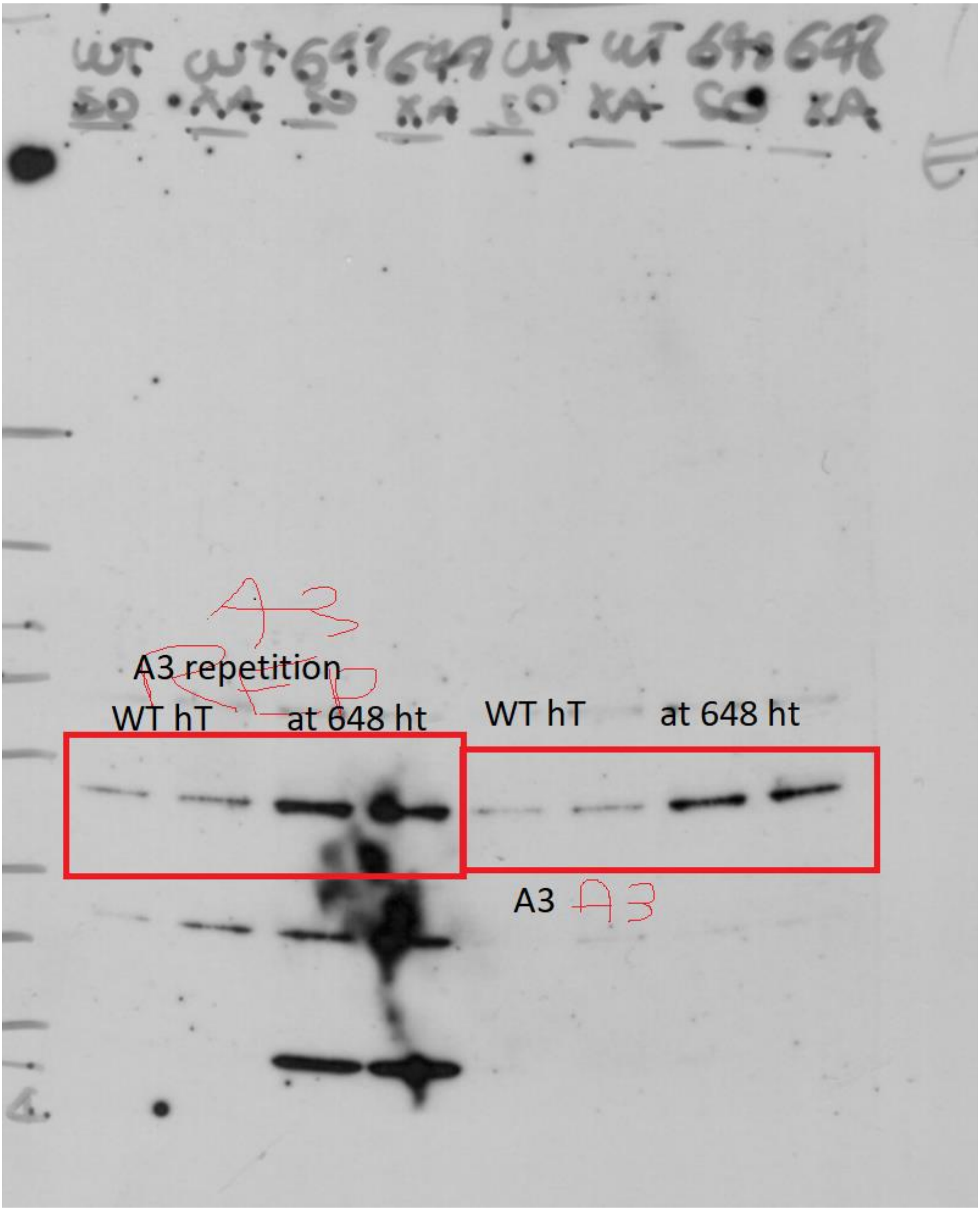

S8O ab2

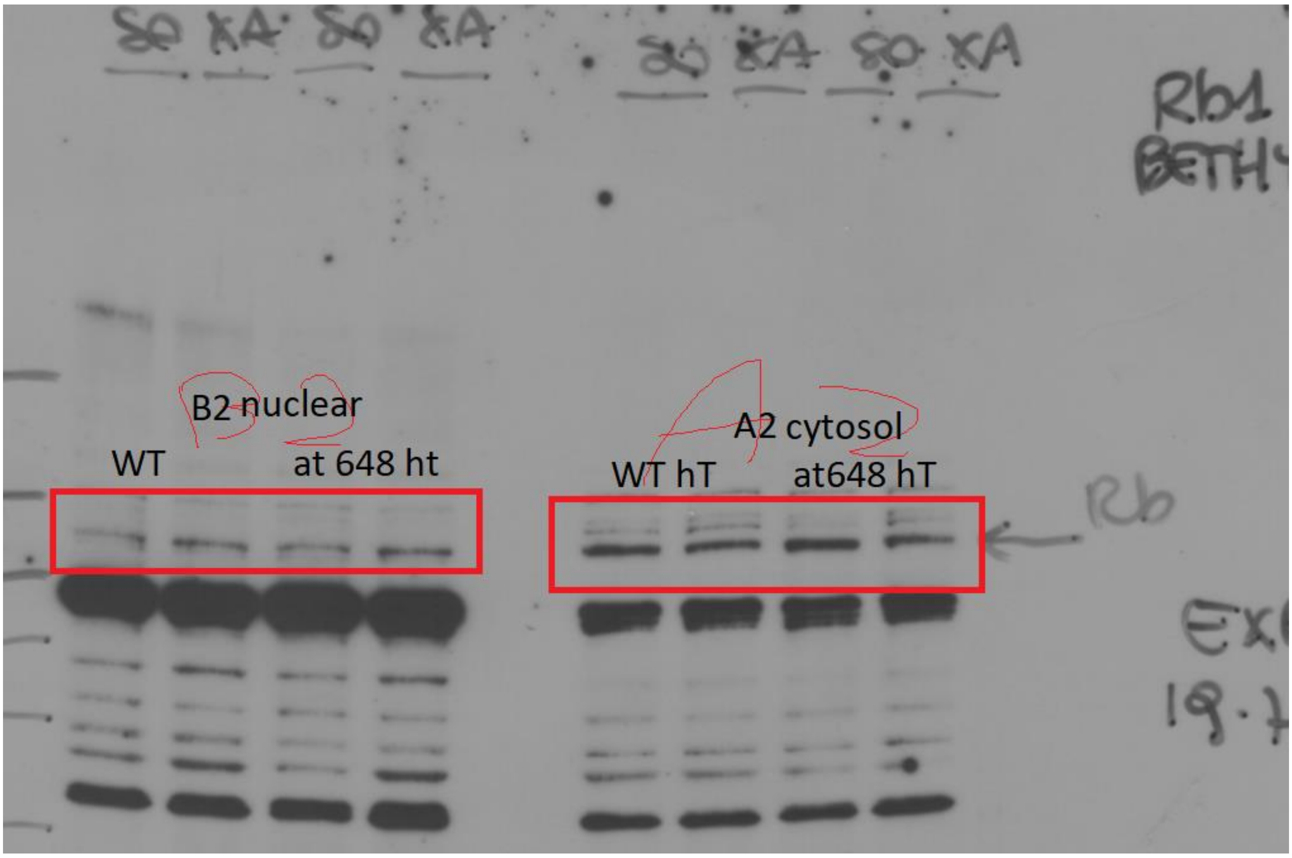

S8O b1 long

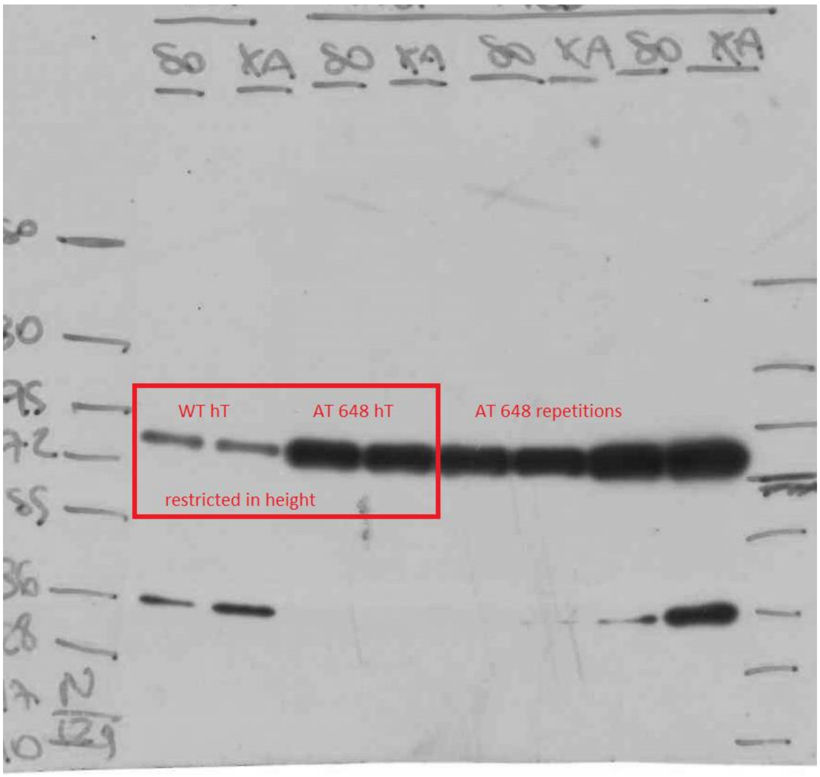

S8O b1 short

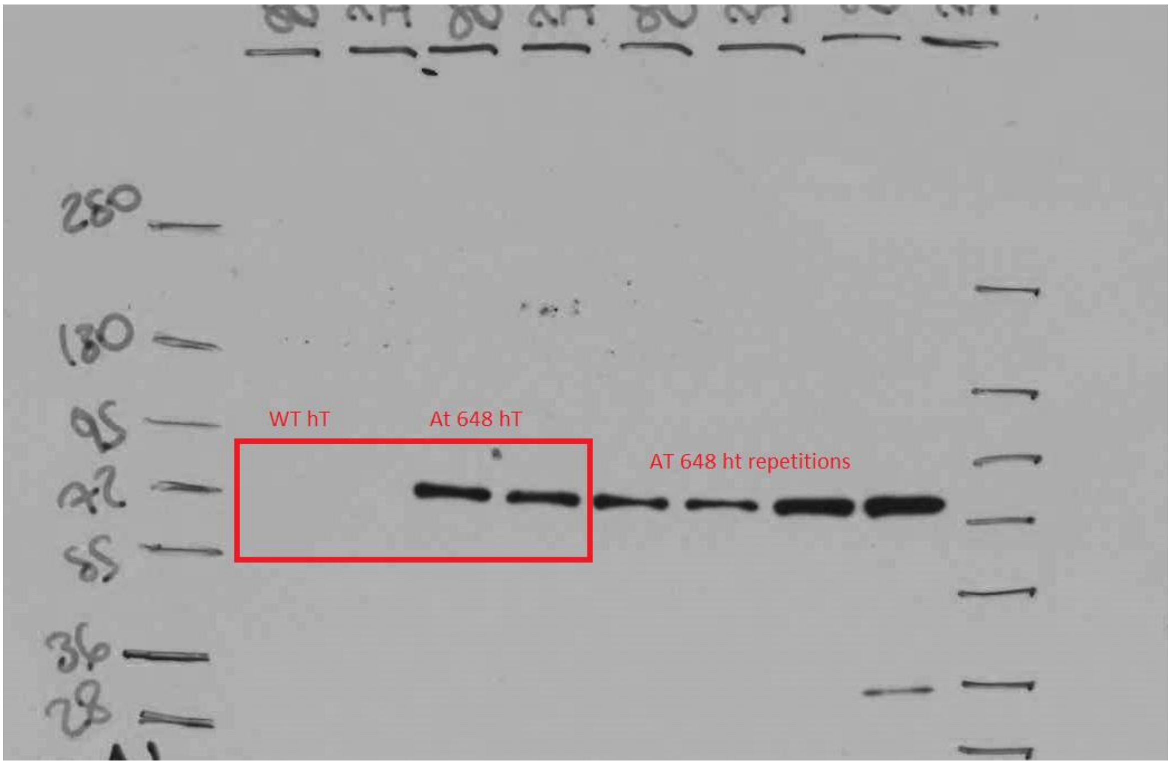

S8O b3 shortl

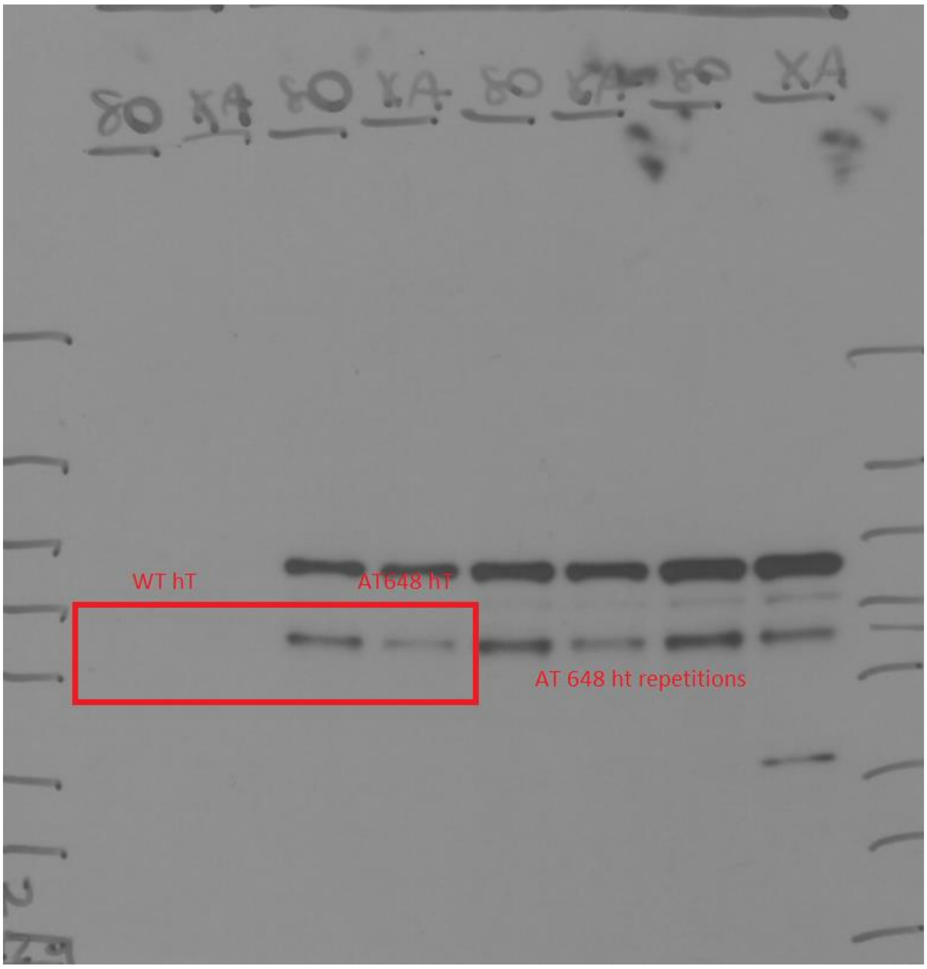

S8O b3 long

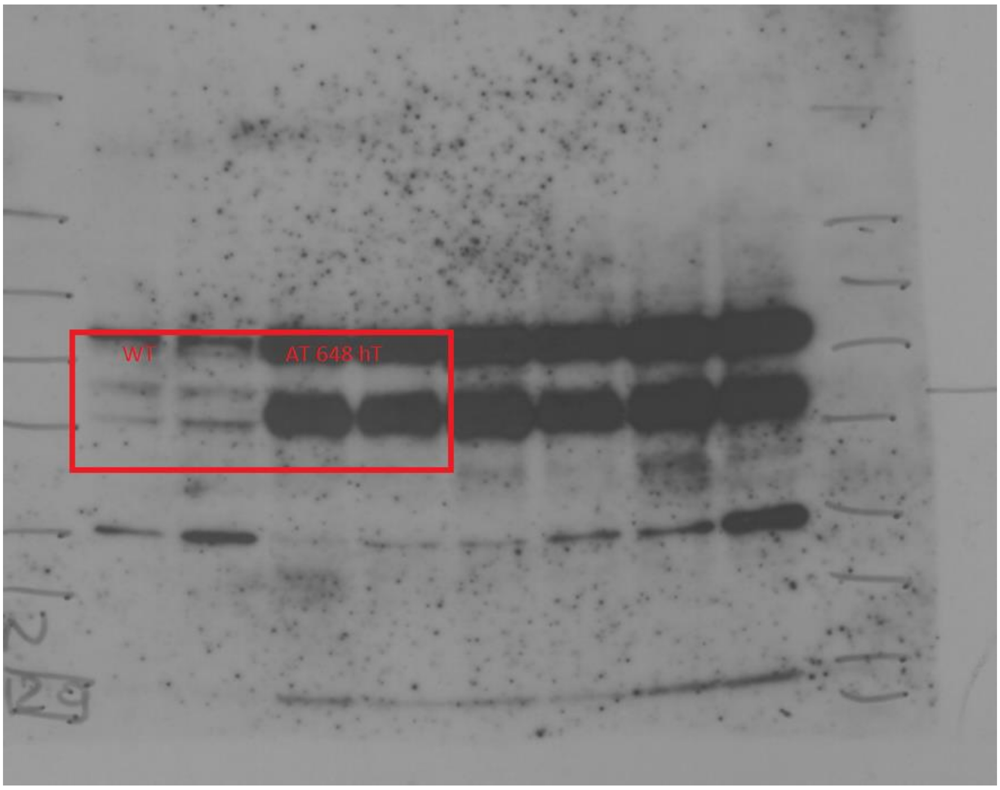

S8O_a1_WT_648

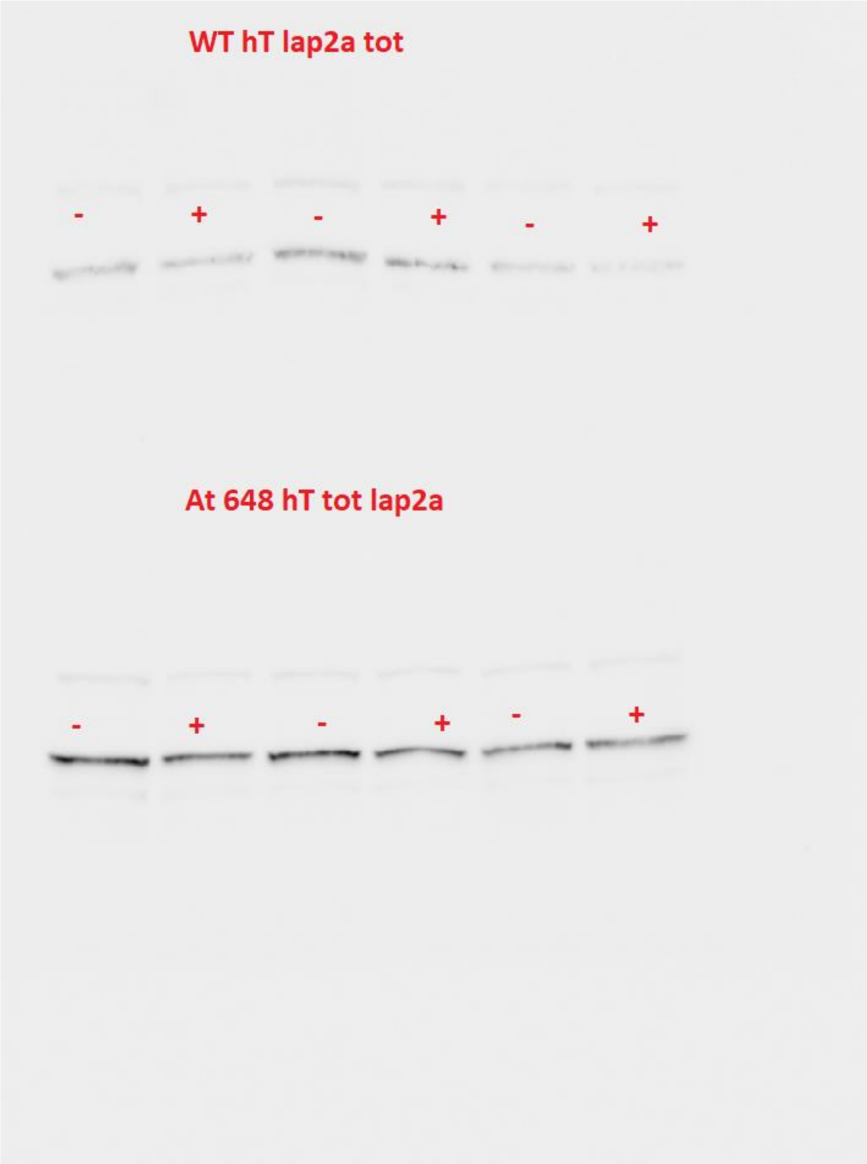

S8O_a2_wt_648

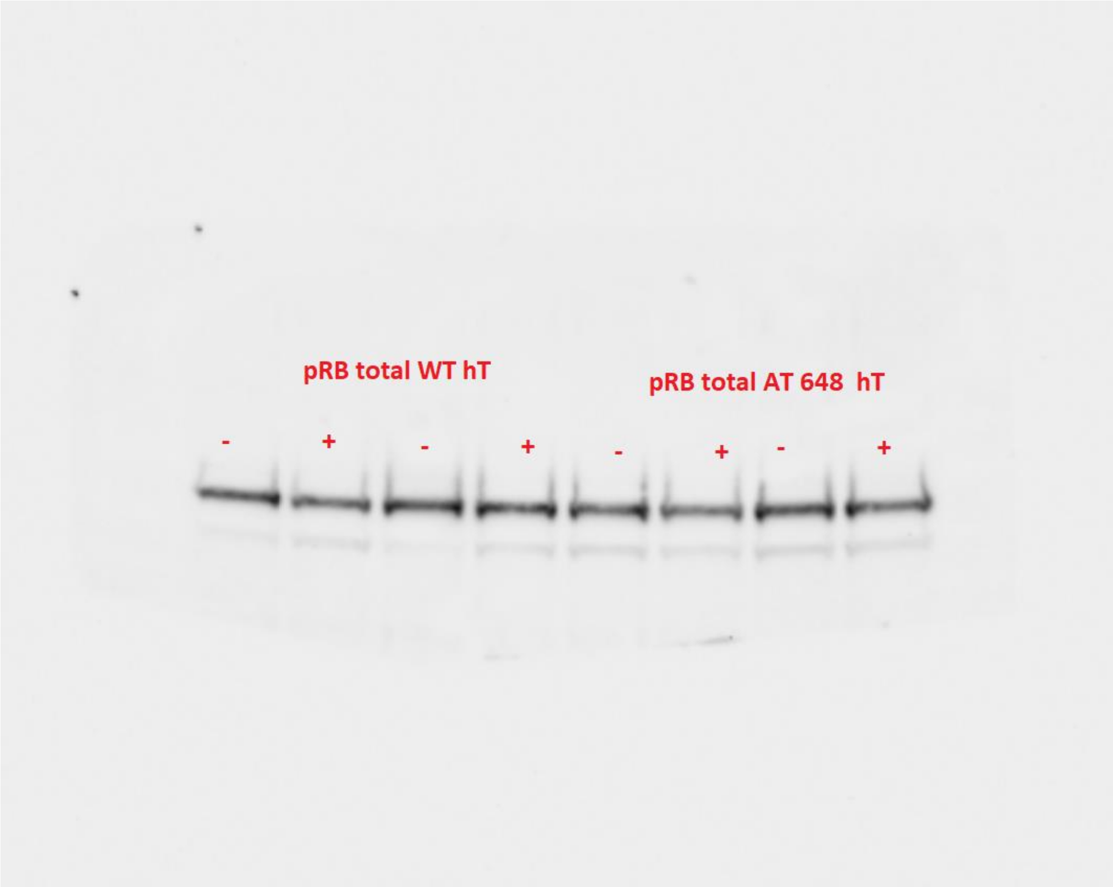

S8O_a3_WT_648

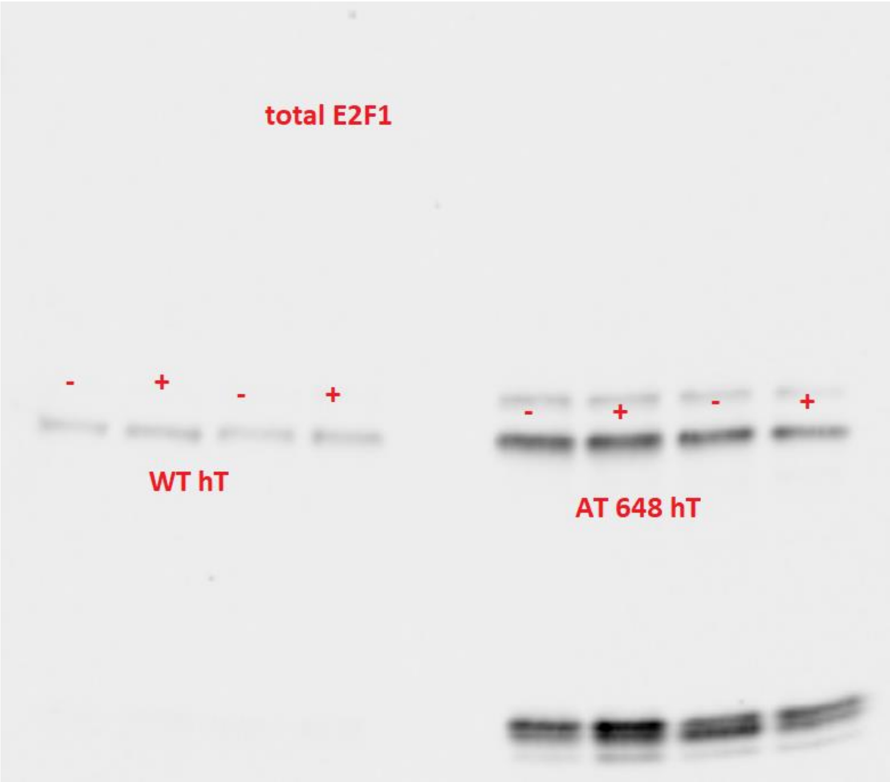

S8O_b1_648

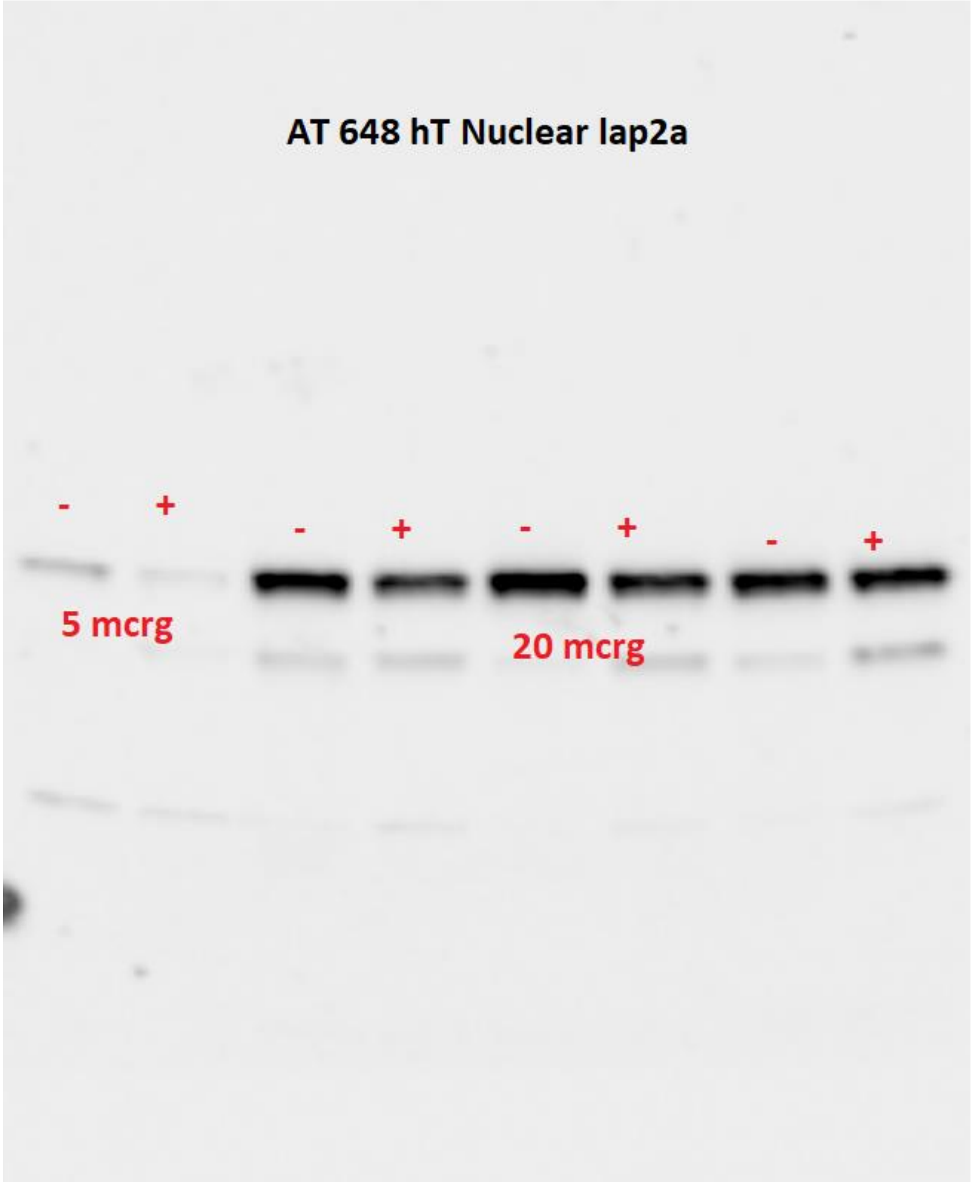

S8O_b1_WT

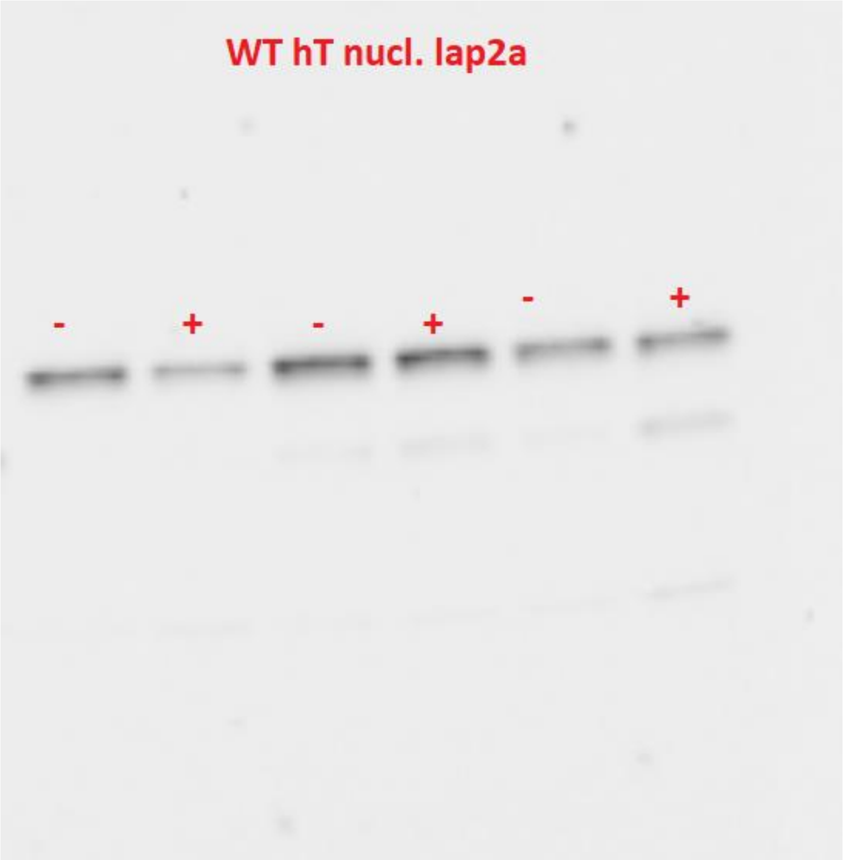

S8O_b2_WT_648

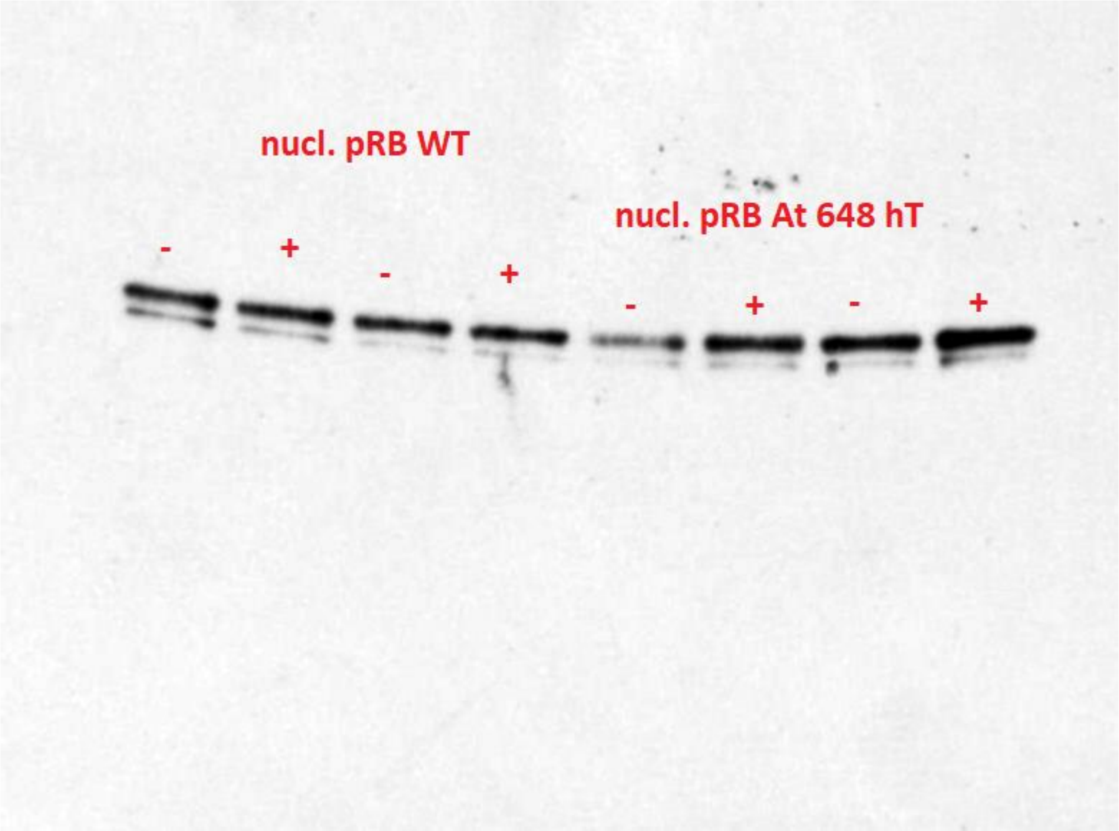

S8O_b3_WT_648

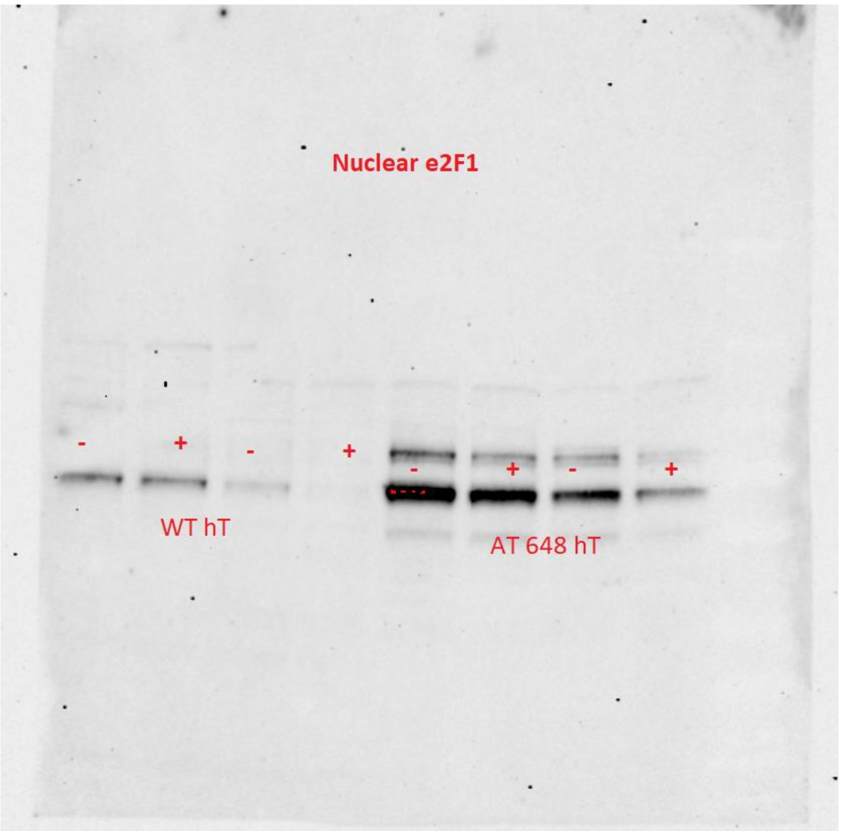

Supplement: Supplementary file 4 — Supplementary figures. [file 41598_2021_89608_MOESM4_ESM.pdf]
